# Chromosome-free bacterial cells are safe and programmable platforms for synthetic biology

**DOI:** 10.1073/pnas.1918859117

**Published:** 2020-03-06

**Authors:** Catherine Fan, Paul A. Davison, Robert Habgood, Hong Zeng, Christoph M. Decker, Manuela Gesell Salazar, Khemmathin Lueangwattanapong, Helen E. Townley, Aidong Yang, Ian P. Thompson, Hua Ye, Zhanfeng Cui, Frank Schmidt, C. Neil Hunter, Wei E. Huang

**Affiliations:** ^a^Department of Engineering Science, University of Oxford, Oxford OX1 3PJ, United Kingdom;; ^b^Department of Molecular Biology and Biotechnology, University of Sheffield, Sheffield S10 2TN, United Kingdom;; ^c^Interfaculty Institute for Genetics and Functional Genomics, University Medicine Greifswald, 17475 Greifswald, Germany;; ^d^Proteomics Core, Weill Cornell Medicine-Qatar, Doha, Qatar

**Keywords:** synthetic biology, SimCells, minimal genome, bacterial therapy, cancer

## Abstract

A type of chromosome-free cell called SimCells (simple cells) has been generated from *Escherichia coli*, *Pseudomonas putida*, and *Ralstonia eutropha.* The removal of the native chromosomes of these bacteria was achieved by double-stranded breaks made by heterologous I-CeuI endonuclease and the degradation activity of endogenous nucleases. We have shown that the cellular machinery remained functional in these chromosome-free SimCells and was able to process various genetic circuits. This includes the glycolysis pathway (composed of 10 genes) and inducible genetic circuits. It was found that the glycolysis pathway significantly extended longevity of SimCells due to its ability to regenerate ATP and NADH/NADPH. The SimCells were able to continuously express synthetic genetic circuits for 10 d after chromosome removal. As a proof of principle, we demonstrated that SimCells can be used as a safe agent (as they cannot replicate) for bacterial therapy. SimCells were used to synthesize catechol (a potent anticancer drug) from salicylic acid to inhibit lung, brain, and soft-tissue cancer cells. SimCells represent a simplified synthetic biology chassis that can be programmed to manufacture and deliver products safely without interference from the host genome.

## Significance

We constructed simple cells (SimCells) whose native chromosomes were removed and replaced by synthetic genetic circuits. The chromosome-free SimCells can process designed DNA and express target genes for an extended period of time. The strategy of SimCell generation is applicable to most bacteria, creating a universal platform for reprogramming bacteria. We demonstrated that SimCells can be designed as safe agents for bacterial therapy through synthesis and delivery of a potent anticancer drug against a variety of cancer cell lines. This showed that the nonreplicating and programmable property of SimCells is advantageous for applications in sensitive environments. The results of this work will both improve our understanding of natural living systems and simultaneously lay the foundations for future advances in synthetic biology.

The aim of synthetic biology is to engineer organisms to perform researcher-designed functions in a safe, reliable, and robust manner, while the objective of living organisms is to survive, which is facilitated by adaptation, evolution, and reproduction. This inherent conflict will jeopardize the performance of synthetic genetic circuits due to the unwieldy complexity and variability of cells (1), unpredictable gene expression, and interference from native gene networks (2, 3), as well as defensive disruption from transposable elements universally present in most organisms (4). The generation of chromosome-free cells (simple cells or SimCells) addresses this issue as without the interference of native gene networks, SimCells should express synthetic genetic circuits in a more predictable manner (5–8).

As SimCells are reprogrammable chassis cells that exist in a boundary state between life and nonlife, SimCells could not only provide insights into the minimal requirements of life but also expand the applicability of bacterial agents to sensitive environments. For example, one application of interest for SimCells highlighted in this study is bacterial cancer therapy (9). As it is known that some bacteria preferentially associate with tumors (10), synthetic biology tools and concepts can be used to further engineer bacteria to sense, target, and deliver anticancer compounds locally to tumors (11–14). SimCells offer an additional advantage as a therapeutic agent as they cannot replicate due to the lack of chromosomal DNA.

In this study, we demonstrated that these chromosome-free SimCells can be readily generated from a variety of bacteria such as *Escherichia coli*, *Pseudomonas putida*, and *Ralstonia eutropha* by inducible chromosomal degradation. Native chromosomes were removed by I-CeuI endonuclease-mediated double-stranded breaks (DSBs) and endogenous nucleases (15). The cellular machinery which remained in the chromosome-free SimCells was able to process various designed genetic circuits and synthesize proteins. It was found that a basal-level expression of the glycolysis pathway significantly extended the functionality of the transcription and translation apparatus and maintained enzymatic activity (e.g., salicylate hydroxylase) in SimCells by regenerating ATP and NADH. As a proof of principle, we demonstrated that SimCells can serve as a safe bacterial therapy agent, converting salicylic acid (SA) into catechol, which was cytotoxic against several malignant cancer cell lines that have low patient survival rates: adenocarcinoma (lung cancer) (16), glioblastoma (brain cancer) (17), and rhabdomyosarcoma (soft-tissue cancer) (18). Hence, SimCells not only have vast potential in elucidating the minimal requirements for life but can also be utilized as biocatalysts, biosensors, and more in sensitive environments.

## Results

### SimCell Generation by Degradation of the Native Chromosome.

SimCells were generated by making several DSBs on the chromosome, which led to chromosomal DNA degradation facilitated by RecBCD helicase-nuclease and other endogenous nucleases (15, 19, 20). DSBs were made via I-CeuI endonuclease, which recognizes a specific 26-base pair sequence (5′-TAACTATAACGGTCCTAAGGTAGCGA-3′) (21). This I-CeuI recognition sequence is naturally present in most bacterial genomes as it is encoded within the conserved *rrl* gene of 23S rRNA (22). Thus, depending on the copy number of 23S rRNA, I-CeuI endonuclease has multiple recognition sites in the genomes of bacteria (seven, six, and three sites in *E. coli*, *P. putida*, and *R. eutropha*, respectively). This particular endonuclease was therefore a strategic choice because it enables a straightforward conversion of most bacteria to SimCells. In addition, there is a very low probability (1 in 4.5 × 10^15^ bp sequences) that this 26-bp recognition sequence would appear in designed genetic circuits or minigenomes.

The creation of multiple DSBs is lethal to bacterial cells; therefore, a strain containing *I-CeuI* cannot survive unless the expression of *I-CeuI* is under very tight control. To choose the optimal genetic circuit to express I-CeuI endonuclease for chromosomal degradation, we screened a variety of tight regulatory systems (23, 24). The TetR family repressors TetR (pJKR-H-TetR) and EilR (pRH121) (*SI Appendix*, Table S1) showed very low basal expression when not induced but a high level of expression when induced (23, 24). Thus, the *I-CeuI* gene was placed under control of the anhydrotetracycline (ATc) and crystal violet inducible TetR and EilR genetic circuits (*SI Appendix*, Figs. S1*B* and S2*A* and Table S1). The TetR system was used in *E. coli*, and the EilR system was used in *P. putida* and *R. eutropha*. When induced, I-CeuI made several DSBs in the chromosomes of these organisms. A DSB is usually repaired through homologous recombination with RecBCD and RecA (25). However, since I-CeuI simultaneously produced several unrepairable DSBs in the chromosome, it resulted in instability and subsequent DNA breakdown by RecBCD and other nucleases (19, 26–29). Based on the gradual loss of DAPI (Movies S1–S3), chromosomes were shown to be removed after the chromosome cutting activity of multiple DSBs and degradation by nucleases (*SI Appendix*, Fig. S3). These chromosome-free cells have now become simple cells or SimCells. A control strain that lacked *I-CeuI* expression preserved the chromosome and did not lose the blue fluorescence of DAPI (Movie S1*B* and *SI Appendix*, Fig. S3) when observed for the same amount of time. The degradation rates of the *E. coli*, *P. putida*, and *R. eutropha* chromosomes were 200, 225, and 147 bp/s, respectively (*SI Appendix*, Table S3). These rates were likely dependent on the number of I-CeuI restriction sites, the size of the genome, and the number of RecBCD molecules. Moreover, the successful degradation of the chromosomes of *E. coli*, *P. putida*, and *R. eutropha* validates the SimCell generation method as being a universal approach for the production of chromosome-free cells from different bacterial genera.

### Functionality and Longevity Design for Characterization of SimCells.

**Fig. 1.**
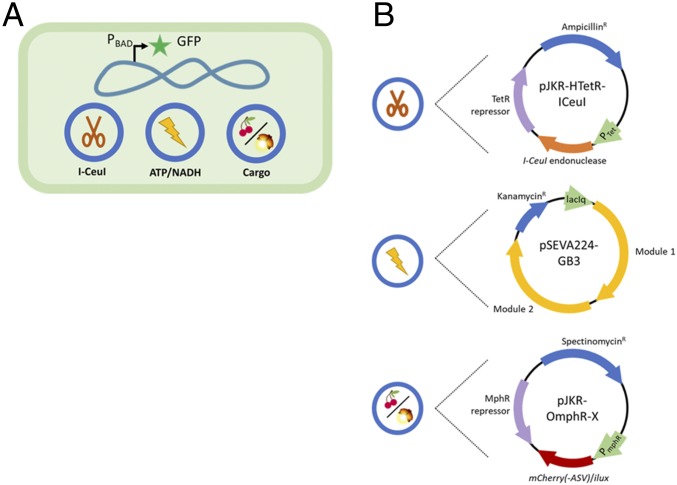
The main features for the constructed SimCell characterized in this study. (*A*) To distinguish SimCells from normal cells, a strain with chromosomal expression of GFP controlled by the arabinose operon was used to indicate the integrity of the chromosome. Three additional plasmids are used to degrade the chromosome, reintroduce glycolysis, and produce protein (mCherry, mCherry-ASV, or the *ilux* operon). (*B*) For SimCell generation (chromosome degradation), pJKR-HTetR-ICeuI (high copy number plasmid) was constructed to put I-CeuI endonuclease production under control of the TetR regulation system which is inducible by ATc. To improve longevity, ATP and NADH generation was supplemented to cells via the glycolysis pathway carried by pSEVA224-GB3 (low copy number plasmid). The glycolysis pathway genes are controlled by the lac system inducible by IPTG. However, the *I-CeuI* and the glycolysis genes were not induced for the majority of experiments presented in this work. Finally, the strain contains variants of pJKR-O-mphR-X (medium copy number plasmid) which produces either mCherry, mCherry-ASV, or luminescence depending on the application. This plasmid utilizes the MphR regulation system which is inducible by erythromycin.

The constructed SimCell needed to possess and demonstrate several properties in order to validate its identity and functionality. Fig. 1*A* is an overview of the elements contained in a parent *E. coli* cell before it becomes a SimCell, and Fig. 1*B* details the attributes of each plasmid. The functions of the three plasmids are controllable chromosome cutting (pJKR-HTetR-ICeuI), ATP and NADH regeneration (pSEVA224-GB3), and cargo expression (pJKR-OmphR-X) (*SI Appendix*, Table S1). To confirm that the cell is chromosome-free, the parent strain contains a chromosomal GFP marker controlled by an arabinose regulation system. Parent cells that still have their chromosomes will produce GFP, while the chromosome-free SimCells will be unable to produce GFP when induced with arabinose.

A gene cluster encoding the entire glycolysis pathway, which produces ATP and NADH, was included in the SimCells. The gene cluster contains 10 genes under control of the IPTG-inducible promoter P_trc_ (30) on the plasmid pSEVA224-GB3 (*SI Appendix*, Table S1 and Fig. S1*C*). The cargo expression plasmid inside the SimCells differed depending on the proof-of-concept experiment being conducted, but they all were controlled by a tightly regulated MphR system encoded by pJKR-O-mphR to produce some product in response to erythromycin induction. The cargo carried on pJKR-OmphR-X was either *mCherry* (*SI Appendix*, Fig. S1*D*), an unstable variant of mCherry (*mCherry-ASV*) (*SI Appendix*, Fig. S1*F*), or the *ilux* operon (*SI Appendix*, Fig. S1*E*). The former established functionality and the latter two elucidated the longevity of SimCells.

The three plasmids (pJKR-HTetR-ICeuI for chromosomal degradation, pSEVA224-GB3 for energy regeneration, and pJKR-OmphR-X for protein production) were transferred to the parent *E. coli* strain BW31003 before *I-CeuI* was induced to generate SimCells. The strains without *I-CeuI* and/or the glycolysis pathway, containing only the vector backbones (pSEVA224 and pJKR-H-TetR), acted as the control groups in this study.

### Quantification of SimCell Generation Efficiency.

The efficiency and efficacy of I-CeuI endonuclease in generating SimCells was characterized using cell viability and flow cytometry to estimate the proportion of SimCells in a mixed population after I-CeuI endonuclease activity. As SimCells no longer contain the chromosome and thus cannot replicate, cultures were plated to see how many parent *E. coli* cells remained in the population by counting the colony forming units (CFU) (Table 1). The strains with the *I-CeuI* gene had CFU/mL about two magnitudes lower compared to its corresponding control strain (no *I-CeuI*). This indicated that I-CeuI endonuclease indeed had an effect on cell viability as the chromosomes would have been degraded (Movies S1*A*, S2, and S3). Based on these results, induced I-CeuI cultures still contained about 1.4 and 3.8% parent cells without and with the glycolysis plasmid, respectively.

**Table 1.** CFU/mL of I-CeuI+ versus I-CeuI- cultures

Strain	CFU/mL
ICeuI^+^ glycolysis^−^	2.67 × 10^6^
ICeuI^−^ glycolysis^−^	1.87 × 10^8^
ICeuI^+^ glycolysis^+^	5.58 × 10^6^
ICeuI^−^ glycolysis^+^	1.46 × 10^8^

Overnight cultures of strains with or without the *I-CeuI* gene (pJKR-HTetR-ICeuI) and with or without the glycolysis pathway (pSEVA224-GB3) were plated out to count the CFU/mL and quantify the number of normal cells remaining in the population. The dilutions that were plated out were normalized to have the same OD_600_. Strains with *I-CeuI* had about 100x fewer colonies than those without *I-CeuI*, demonstrating the adverse effect of *I-CeuI* on cell viability due to chromosomal degradation.

**Fig. 2.**
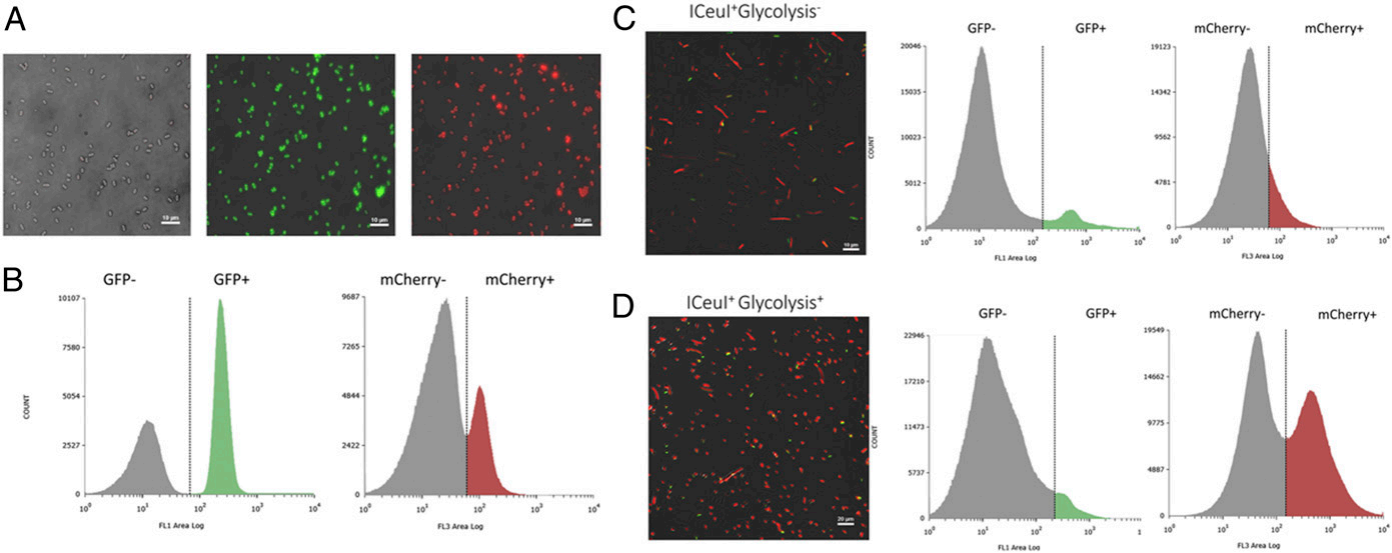
FL1 (GFP) and FL3 (mCherry) channel histograms of flow cytometry analyses and corresponding microscopy images. (*A*) Fluorescence microscopy images of a control strain (no *I-CeuI*) to show integrity of the GFP and mCherry biosensors. The parent strain (I-CeuI-) was (*Left*) not induced, (*Center*) induced for GFP expression, or (*Right*) induced for mCherry expression. (*B*) FL1 (GFP) and FL3 (mCherry) channel histograms had distinct populations which were used to define no expression and GFP expression (SimCell identity gates) or mCherry expression (protein production) when two populations were not obvious in the experimental groups. (*C*) The I-CeuI+ strain without the glycolysis pathway (pSEVA224-GB3) contained a mixed population of SimCells and normal cells. Based on the two somewhat distinct populations, about 91.32% of cells are SimCells (GFP-). Of the SimCell population, only 13.18% produced mCherry. (*D*) In the I-CeuI+ strain with the glycolysis pathway (pSEVA224-GB3), about 94.36% of cells are SimCells (GFP-), a slightly higher proportion compared to the strain without glycolysis. Of the SimCell population, 56.20% produced mCherry, a large improvement compared to the strain without glycolysis.

Flow cytometry was used to quantify chromosome-free SimCells by using the arabinose-inducible *gfp* circuit on the chromosome of parent cells. Fluorescence microscopy showed that both the AraC and MphR regulation systems were tight, and basal expression was not observed from uninduced strains. When induced, there was clear and obvious expression of GFP and mCherry (Fig. 2*A*). Control cell cultures (no *I-CeuI*) with and without arabinose or erythromycin induction were mixed to define GFP or mCherry expression boundaries when there were not distinct populations (Fig. 2*B*). Based on flow cytometry analysis, the majority of the *I-CeuI*–induced culture were SimCells: 91.32% of the population in strains without glycolysis and 94.36% with glycolysis (Fig. 2 *C* and *D*). Assuming the rest were parent cells (8.68 and 5.64%), estimations of the parent cell population remaining in *I-CeuI*–induced cultures were slightly higher with flow cytometry versus cell plating experiments. This discrepancy could be due to ambiguity of GFP expression as determined by flow cytometry.

It was unexpected to observe a substantial population of parent cells remaining in an induced *I-CeuI* population as the seven DSBs made on the *E. coli* chromosome should have been very destructive. However, the bacteria were still able to deploy their arsenal of survival strategies to deactivate the *I-CeuI* gene in later generations, thereby restoring growth fitness and accounting for the existence of parent cells; this phenomenon is explored further in another study (4). It also reinforces the idea that there is a need for a stable and robust chassis cell for synthetic biology. By lacking a genome, SimCells are a step forward in addressing this need as they avoid the possibility of genetic drift and evolvability (31, 32).

### Expression of Designed Genetic Circuits by SimCells.

Once it was established that chromosome-free SimCells could be generated, we sought to prove our premise that plasmid DNA or minigenomes could prompt SimCells to express designed genetic circuits. Flow cytometry was also used to determine the population of SimCells that were expressing mCherry (pJKR-O-mphR). As done previously, a control strain (no *I-CeuI*) was either not induced or induced for mCherry expression to define expression boundaries when distinct populations were not observed (Fig. 2*B*). Using the previously established GFP expression gates, the SimCell population (no GFP) was isolated. Of the SimCell population, it was estimated only 13.18% of those without glycolysis were producing mCherry, but this proportion considerably increased to 56.20% with the reintroduction of glycolysis (ATP and NADH generation) (Fig. 2 *C* and *D*). A proportion of SimCells could not produce mCherry. The restoration of just one metabolic pathway (glycolysis) was likely not sufficient to obtain a fully functional SimCell population. However, this large improvement in SimCell performance shows that it is important to reintroduce metabolic pathways in minimal cells to provide ATP and NADH for cellular processes to function.

**Fig. 3.**
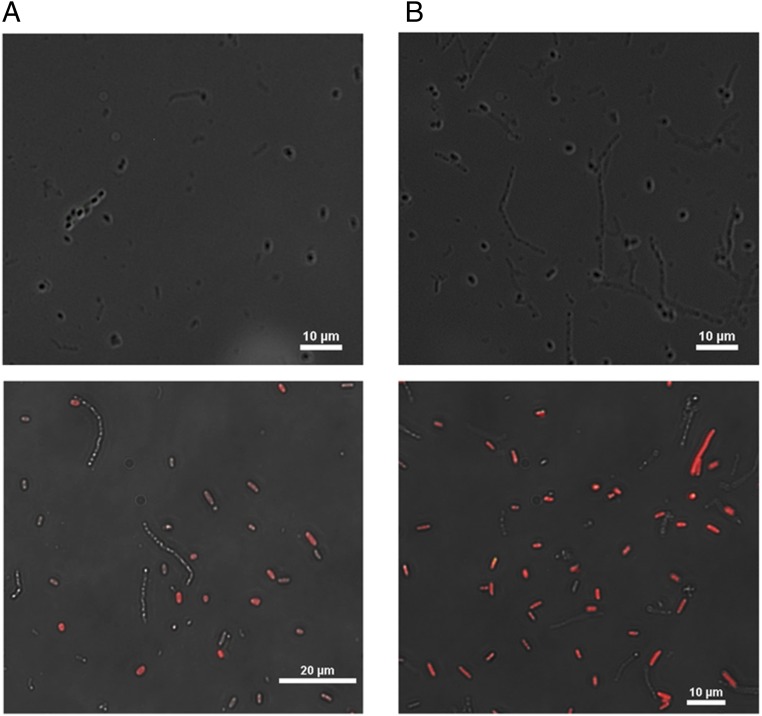
I-CeuI+ cultures were treated with the drug D-cycloserine to kill parent cells to yield a pure SimCell culture. (*A*) SimCell culture without glycolysis (*Top*) not induced and (*Bottom*) induced for GFP and mCherry production. (*B*) SimCell culture with glycolysis (*Top*) not induced and (*Bottom*) induced for GFP and mCherry production. No cells fluoresced green, which meant D-cycloserine was effective in killing parent cells and producing a pure SimCell culture. A higher proportion of SimCells (no GFP expression) expressed mCherry when supplemented with glycolysis (pSEVA224-GB3).

Fig. 2 *C* and *D* also show fluorescence microscopy images of strains with *I-CeuI* induced for GFP and mCherry expression. Cells that are expressing GFP still have an intact chromosome so, thus, are not SimCells. Cells that are only fluorescing red are SimCells that are producing mCherry. The results from flow cytometry and fluorescent microscopy images are consistent, indicating that SimCells with pSEVA224-GB3 (encoding glycolysis pathway mediated ATP and NADH generation) enhances functionality (Figs. 2 and 3). There are more SimCells producing mCherry as glycolysis was able to generate ATP and NADH. These images (Fig. 2 *C* and *D*) also show that flow cytometry can be used to estimate the proportion of SimCell production and to quantify the efficiency of an inducible genetic circuit in SimCells.

To confirm that these red-only cells are nonreplicating SimCells producing protein, cells were fixed in LB agar with the inducers arabinose and erythromycin and allowed to grow overnight. The control strain (no *I-CeuI*) (*SI Appendix*, Fig. S4*A*) grew colonies that dominated the agar, and there was expression of both GFP and mCherry. In contrast, a strain with *I-CeuI* (*SI Appendix*, Fig. S4*B*) contained SimCells which have lost the ability to replicate. While there were a few parent cells that avoided chromosomal degradation and replicated to form colonies that expressed GFP and mCherry, there were more individual SimCells that were only expressing mCherry. This confirmed that cells which were not expressing GFP were chromosome-free, nonreplicating cells. Movie S4 shows the progression of SimCell generation and subsequent protein production. Cells containing chromosomal DNA were stained with DAPI and fixed in PBS agar containing I-CeuI endonuclease, GFP, and mCherry inducers. After induction of I-CeuI expression, the degradation of the chromosome was triggered. This is evidenced by the loss of blue fluorescence as the chromosomal DNA degraded into nucleotides. The newly formed SimCell then started producing red fluorescence (mCherry). These observations demonstrated that SimCells were indeed producing mCherry and that mCherry was not an artifact from synthesis performed by the parent cell.

In summary, these results show that the chromosome-free SimCells could still produce protein when instructed by a genetic circuit, and this activity can be enhanced by reintroducing the glycolysis pathway to generate ATP and NADH.

### Purification of SimCells.

As shown by the flow cytometry and cell plating experiments, SimCell generation was not fully efficient after I-CeuI endonuclease activity. In order to elucidate distinct features and characteristics of SimCells, they needed to be purified from parent cells. Previous methods of purification were attempted or considered (FACS, density or charge-based cell sorting, magnetic nanoparticles, etc.); however, they were either uneconomical or ineffective. D-cycloserine has previously been used to purify minimal cell cultures (33), so it was evaluated for its efficacy in killing actively dividing parent cells that remained in *I-CeuI*^+^ cultures. Table 2 shows the CFU/mL of strains over time after application of D-cycloserine at time 0 and 1.5 h. In the presence of D-cycloserine, actively dividing cells will not survive, because cell wall biosynthesis is inhibited by the drug. This means the cell wall cannot sustain increasing cellular volume, resulting in osmotic lysis (34–36). Although D-cycloserine was unable to kill all of the cells in the control strain, in an *I-CeuI*^+^ strain parent cells were effectively eliminated, yielding a pure SimCell culture. This can also be seen in Fig. 3 in which no cells are expressing GFP, indicating that they lack a chromosome and are thus SimCells. As SimCells were purified first and then induced for mCherry expression, these results are also evidence that SimCells produced protein and it was not residual expression from parent cells. These images also highlight the benefit of glycolysis as it allowed more SimCells to produce mCherry (Fig. 3).

**Table 2.** Purification of SimCells with D-cycloserine

Time (h)	ICeuI^−^glycolysis^−^	ICeuI^+^ glycolysis^−^	ICeuI^−^ glycolysis^+^	ICeuI^+^ glycolysis^+^
0	1.8 × 10^9^	3.5 × 10^5^	1.0 × 10^9^	3.08 × 10^5^
1.5	1.1 × 10^9^	4.1 × 10^3^	6.9 × 10^8^	2.7 × 10^3^
5	1.3 × 10^7^	7.2 × 10^2^	4 × 10^7^	7.5 × 10^2^
24	1.6 × 10^7^	0	8.3 × 10^4^	0

Cultures were treated with 200 µg/mL D-cycloserine at time 0 and 1.5 h to kill normal cells to yield a pure SimCell culture. Plate counts over time estimated the CFU/mL and efficacy of the drug. Several dilutions were plated to ensure normal cells were killed. D-cycloserine effectively killed normal cells in strains with *I-CeuI*, indicating that it was an effective drug.

Further optimization of D-cycloserine concentration ensured pure SimCell populations in the following experiments and excluded the contribution made by any remaining parent cells in SimCell populations. After a screen of D-cycloserine concentrations, 25 µg/mL was determined to be the point where parent cells were killed in the culture evidenced by little to no increase in OD600 but also permitted protein production or other activities in SimCells. By using D-cycloserine which targets active, parent cells, we were able to distinguish activity from SimCells on a larger, culture-level scale for further characterization.

### Shelf Life of SimCells.

In order to establish the applicability of SimCells, it was necessary to characterize how long they could be maintained in storage. SimCells were purified using D-cycloserine from strains containing the mCherry expression plasmid and with or without the pSEVA224-GB3 plasmid encoding the glycolysis pathway. Aliquots of purified SimCell culture were then stored at either 4 or −80 °C with glycerol. At 1 wk, 1 mo, and 5 mo after storage, SimCells were induced for mCherry production. *SI Appendix*, Fig. S5, shows that SimCells can be preserved for at least 5 mo. As expected, more SimCells can produce mCherry with the glycolysis pathway than those without, although the number of SimCells that can produce mCherry decreased after they were stored. In general, it seemed that SimCells preserved at 4 °C performed better than those stored at −80 °C in terms of inducible mCherry expression from pJKR-O-mphR. There is some evidence that suggests storage at subzero temperatures and subsequent thawing can cause metabolic injury to cells (37) and affect growth kinetics (38). In the case of SimCells, it is likely that at 4 °C, cellular processes were quiescent rather than shut down at −80 °C, and thus, cells were more readily reactivated and recovered.

### Longevity of SimCells.

**Fig. 4.**
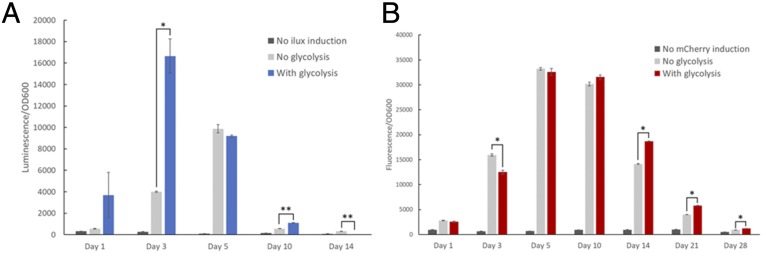
Longevity of the cellular machinery within SimCells (protein synthesis, regulation, and degradation) was elucidated by indirectly tracking the cellular availability of ATP/NADH. (*A*) SimCells could produce luminescence for about 10 d, indicating they had sufficient energy currency to sustain functionality for a significant amount of time. The strain with the glycolysis pathway had a significant energy advantage over the strain without glycolysis in the early days. However, this difference in luminescence production leveled off after day 5. Unpaired (independent) *t* tests were performed: *P < 0.005, **P < 0.001. (*B*) Fluorescence detected from unstable mCherry stopped increasing after 10 d, which is likely the point where transcription and translation did not have enough energy to be operational. This corresponds to the availability of ATP as suggested by luminescence production (A). SimCells had detectable levels of unstable mCherry for a long time (28 d) because the activity of proteases is ATP-dependent. Unpaired (independent) *t* tests were performed: *P < 0.001. Data show means ± SE, *n* = 4.

We considered longevity as the functionality of cell transcription and translation machinery. To characterize the longevity of energy reserves, the production of bioluminescence by purified SimCells via the *ilux* operon was used as an indicator. This is because ATP and/or NADH are needed to facilitate the conversion of FMNH2 and -CHO to FMN and -COOH by enzymes (fatty acid reductase and flavin oxido-reductase) in order for luciferase (*luxAB*) to produce bioluminescence (39). Thus, the *ilux* operon was integrated into the pJKR-O-mphR plasmid (*SI Appendix*, Fig. S1*E*) and introduced to SimCells. Bioluminescence was induced, and its production was tracked in SimCells over time (Fig. 4*A*). Bioluminescence production reached the highest levels around day 3 but decreased thereafter. SimCells seemed be significantly starved of ATP/NADH around day 10, and only background levels of luminescence were detected by day 14. Interestingly, the readdition of the glycolysis pathway made less of an impact on SimCell performance than anticipated. It could be that the enzymes needed for glycolysis remained in suitable concentrations before the SimCells were generated. Therefore, even without the glycolysis plasmid, SimCells could still produce comparable levels of luminescence. It is likely that with the glycolysis plasmid, the higher than average concentration of enzymes allowed an initial burst of increased activity (as seen from day 1 to 3), but this benefit to the cells was eventually lost as the cost of making the 10 proteins in the glycolysis pathway conferred a significant energy burden. This pattern of behavior was also described by our mathematical model (*SI Appendix*, Fig. S7*D*).

Transcription and translation take up a large part of the cell’s energy budget (40) and are important indicators of cellular machinery and protein processes (synthesis, regulation, and degradation), so it is important to evaluate how long the SimCell is able to maintain these processes. To estimate the longevity of the transcription and translation apparatus, as well as the function of degradation enzymes in SimCells, an unstable version of mCherry-ASV was created by fusing a *ssrA* protease tag (VSAAYNEDNAAPR) (41) to the *mCherry* gene, which possessed a shorter half-life time (*SI Appendix*, Fig. S6 and Movie S5). The *mCherry-ASV* reporter gene served as an indication that the major players in protein metabolism such as RNA polymerases, ribosomes, and proteases were still active. Fig. 4*B* shows mCherry-ASV expression over time by purified SimCells with or without glycolysis. Levels of fluorescence reached their highest around day 5 and started to decrease around day 14, and only background levels of fluorescence were detected after 28 d. The benefit of reintroducing glycolysis is not obvious from these results. The protein metabolism machinery seemingly lasted longer than the ATP available in the cell (Fig. 4*A*), but we must also consider the fact that the ClpXP and ClpAP proteases which degrade the *ssrA*-tagged mCherry-ASV (42) require ATP to function (43). Therefore, the point at which fluorescence levels no longer increase (day 5 to 10) is likely when the protein machinery started to lose functionality.

In summary, SimCells have a longevity of about 10 d in terms of access to an energy source and functionality of cellular machinery for the expression of genetic circuits and facilitation of metabolic processes. Therefore, SimCells could be robust enough to remain active and functional for most applications in medicine or biomanufacturing.

### The Trade-Off Between Energy Generation and the Cost of Gene Expression.

The glycolysis pathway generates valuable resources such as ATP and NADH, but the expression of glycolytic genes is energetically costly (*SI Appendix*, Fig. S7 *A* and *B*). Hence, there is a delicate balance between energy generation and gene expression. A mathematical model was developed to simulate this trade-off (Eqs. **1**–**4** and *SI Appendix*). The 10 genes that encode the glycolysis pathway are controlled by a P_trc_ promoter and the LacI repressor, which has leaky expression due to the poor binding kinetics of LacI (*SI Appendix*, Fig. S9). The overexpression of glycolysis by IPTG induction in SimCells was so costly and energy-consuming that SimCells were unable to allocate resources to express other genes such as the *ilux* operon or *mCherry-ASV* (*SI Appendix*, Figs. S7 and S8). In contrast, leaky expression or IPTG induction of glycolytic genes in parent cells can assist bioluminescence and mCherry production in SimCells in the first 3 d. However, this benefit is lost after 3 d due to the cost of making glycolytic proteins, and the performance is similar to SimCells that did not contain the glycolysis pathway (*SI Appendix*, Figs. S7 and S8). The mathematical simulation also conceptually supports this observation (*SI Appendix*, Fig. S7 *D* and *F*).

### Proteomics Revealed Changes in Global Regulation of Proteins in SimCells.

We knew that the consequences of I-CeuI endonuclease activity may not be limited to chromosomal degradation. Hence, proteomics analysis was done in a related study (4) to investigate the changes in protein abundances after I-CeuI endonuclease activity. The *E. coli* DH5α strain with no vector or with an empty vector backbone were used as controls, and DH5α with the *I-CeuI* gene was designated as the experimental, SimCell condition. It was found that indeed the protein expression profile of the *I-CeuI*+ culture was almost opposite to that of the control groups (*SI Appendix*, Fig. S10*A*).

**Fig. 5.**
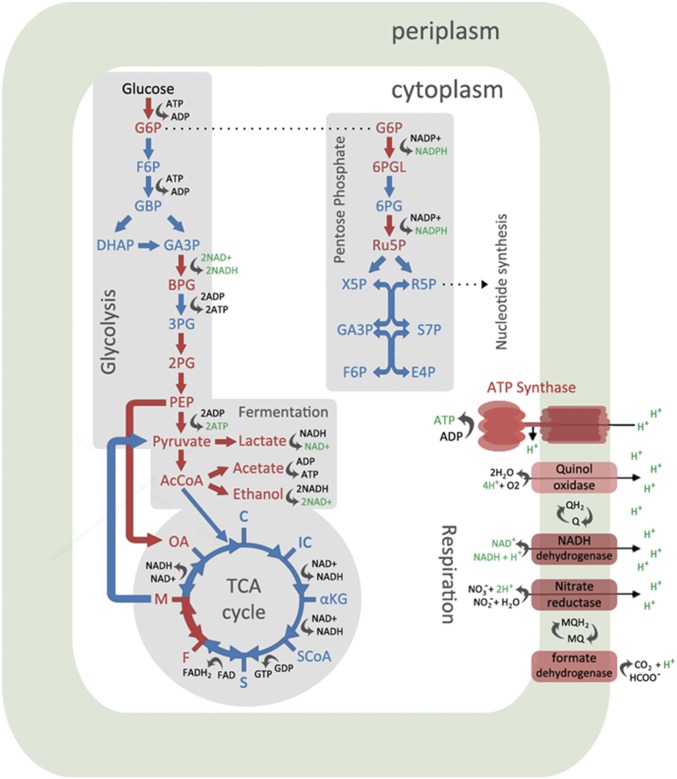
A graphical summary of changes in energy and metabolism in SimCells (I-CeuI+ strains). Elements in red indicate up-regulation of proteins, while elements in blue indicate down-regulation of proteins. Cells favored sections of the glycolysis and pentose phosphate pathway that produced ATP/NADH, while the sections that consumed ATP were down-regulated. The TCA cycle was largely shut down. Fermentation and respiration regenerated NAD+ to be fed back to the glycolysis pathway. The complexes that were up-regulated to generate the proton gradient are shown (other complexes were down-regulated). The resulting proton gradient should drive ATP synthase to produce ATP for the energy-starved cell.

When we dissected the results of specific cellular processes, the same trend of distinct *I-CeuI* protein expression profiles was observed. In terms of energy and metabolism (*SI Appendix*, Fig. S10*B*), a SimCell-induced population up- or down-regulated certain genes to maximize cellular resources to make ATP (energy currency) during a state of energy starvation. Sections of the glycolysis and pentose phosphate pathways that generated ATP and NADH were up-regulated, while sections that consumed ATP were down-regulated (Fig. 5). It is also found that the TCA cycle was largely shut down. This enhanced carbon metabolic response to energy starvation was also observed in other studies where the cell experienced DNA damage and launched a stress response (44). With the deletion of the chromosome, we reduced the functional space of the cell’s metabolic network (45), which resulted in a less complex interactome (the whole set of molecular interactions) and streamlining of the cellular processes in SimCells.

The most obvious phenotypic effect caused by *I-CeuI* is the presence of longer cells, indicating a change in the normal process of cell division (*SI Appendix*, Figs. S3 and S5). A typical *E. coli* is about 2 µm in length, but strains with *I-CeuI* have produced cells that can be 20 µm or longer (Figs. 2 and 3 and *SI Appendix*, Fig. S4*B*). These filamentous cells have been observed before in cells that have launched an SOS response due to DNA damage (*SI Appendix*, Fig. S10*D*) as essential genes for cell division are often shut down by SOS genes (46). However, the SOS response is subsequently silenced after attempted DNA repair (47, 48). Other studies have observed that when FtsZ is down-regulated or mutated in parent cells, cells can reach up to 750 µm in length and are still metabolically competent and will synthesize DNA (49). This preservation of functionality is also supported by our results as the longer SimCells could still produce mCherry (Figs. 2 and 3 and *SI Appendix*, Fig. S4*B*).

The degradation of the chromosome caused by DSBs made by I-CeuI endonuclease launched a dramatic cellular response that affected processes such as cell division, protein metabolism, and ATP generation. Proteomics analysis indicated which processes were prioritized, kept on standby, or shut down and which cellular machineries were present in SimCells, providing insight into the design of genetic circuits or minigenomes.

### SimCells as Safe and Anticancer Delivery Agents.

**Fig. 6.**
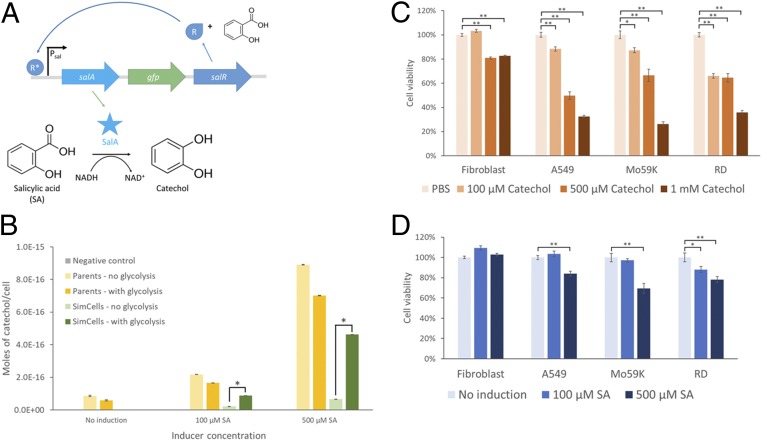
SimCells as a biocatalyst. (*A*) Schematic of the genetic circuit in pSalAR-GFP that produces catechol from SA. When SA is present it combines with SalR to yield an active form, SalR*, which then initiates transcription of *salA* and *salR* (positive feedback). SalA or salicylate hydroxylase then converts SA to catechol in the presence of NADH. There is some basal expression from P_sal_, so low levels of SalA and SalR are already present without induction. (*B*) Quantification of catechol produced by parent cells and SimCells using LC. Moles of catechol produced per cell by strains with pSalAR-GFP or pSalA_Km_xylR (negative control, defective *salA*) were quantified after induction with different concentrations of SA. Strains labeled “no glycolysis” or “with glycolysis” have pSEVA224 or pSEVA224-GB3, respectively. Strains labeled “Parents” or “SimCells” have pJKR-H-TetR or pJKR-HTetR-ICeuI, respectively. More catechol was produced with higher concentrations of inducer. In SimCells, the addition of the glycolysis pathway dramatically improved catechol production. An unpaired (independent) *t* test was performed: *P < 0.001. Data show means ± SE, *n* = 3. (*C*) Anticancer effect of catechol. Fibroblasts and cancer cell lines A549, Mo59K, and RD were treated with different concentrations of catechol (PBS was the control), which resulted in a significant decrease in cell viability of cancer cells. A paired (dependent) *t* test was performed for cell viability assays: *P < 0.005, **P < 0.001. Data show means ± SE, *n* = 6. (*D*) Anticancer effect of catechol produced by SimCells. Fibroblasts and A549, Mo59K, and RD cancer cells were incubated with SimCells induced with different concentrations of SA to produce catechol. There was a significant decrease in cell viability when SimCells were induced to produce the anticancer drug catechol. SimCells induced with 500 μM SA resulted in the largest decrease in cancer cell viability. A paired *t* test was performed for cell viability assays: *P < 0.05, **P < 0.005. Data show means ± SE, *n* = 6.

Catechol (1,2-dihydroxybenzene) is a naturally occurring compound in fruits and vegetables and a moiety of caffeic acid and catechin (tea) (50). Catechol has previously been shown to have selective cytotoxic effects by inducing apoptosis in lung and brain cancer cell lines (51, 52), but it is not harmful to normal cells (e.g., fibroblasts). Taking advantage of the anticancer properties of catechol, SimCells carrying the pSalAR-GFP plasmid were used to convert SA into catechol (53, 54) and export this product to kill cancer cells (Fig. 6*A* and *SI Appendix*, Fig. S1*G*).

The plasmid pSalAR-GFP contains a positive autoregulation genetic circuit, which produces SalA (salicylate hydroxylase) and SalR when induced by SA (Fig. 6*A* and *SI Appendix*, Fig. S1*G*). SalR is a transcriptional regulatory protein and a LysR-type transcriptional regulator (LTTR) family protein that transitions to its active form in the presence of SA or aspirin and acts as an activator for its own promoter P_sal_, effectively forming a positive feedback loop (55, 56). The salicylate-inducible Psal-SalR regulation system has been optimized in *E. coli* (55). SA can activate the P_sal_ promoter within a range of 0.05 to 500 µM and activate the genetic circuit in under an hour (55, 56). SalA catalyzes the conversion of SA to catechol by consuming NADH (53, 57). This plasmid was transferred to *E. coli* parent cells and SimCells (later purified) with and without the glycolysis pathway (pSEVA224-GB3). Liquid chromatography (LC) analysis (Fig. 6*B*) showed a dramatic increase in catechol production by parent cells and SimCells when induced with higher concentrations of SA. This was due to the positive feedback nature of the genetic circuit. SimCells with the glycolysis pathway were able to make comparable amounts of catechol to the parent cell counterparts when induced with 500 µM SA (Fig. 6*B*). The significant improvement of catechol production in SimCells containing the glycolysis pathway can be attributed to the dependency on NADH in salicylate conversion into catechol (57). It suggests that the glycolysis pathway made a large contribution to the conversion of SA to catechol. Given that the SimCell population was 8 × 10^8^ cells per mL, each SimCell produced about 5 × 10^−16^ moles of catechol in 24 h from 500 µM SA. This amount of catechol being produced in the small volume of a bacterial cell (about 1 µm^3^) would have had a high local concentration of catechol, about 500 mM, which subsequently diffused out of the SimCells (*SI Appendix*, Fig. S11). If SimCells were further engineered to target and attach to the surface of cancer cells, the cytotoxic properties of catechol would be enhanced with local delivery.

Screens showed that catechol significantly decreased the viability of A549 (lung), Mo59K (brain), and RD (soft-tissue) cancer cell lines at concentrations as low as 100 µM but had less of an adverse effect against normal cells like fibroblasts (Fig. 6*C*). Additionally, SimCell control, SA (the substrate), and D-cycloserine (to maintain SimCell purity) did not cause the death of four cell lines (fibroblasts, A549, Mo59K, and RD) (*SI Appendix*, Fig. S12), which means a decrease in cell viability could be attributed to the cytotoxic effects of catechol. These cell lines were subsequently incubated with SimCells containing the glycolysis pathway and pSalAR-gfp-full plasmids in various SA concentrations. SimCells induced with 500 µM SA for catechol production significantly lowered the cell viability and confluency of all cancer cell lines but did not kill fibroblasts (noncancerous cells) (Fig. 6*D* and *SI Appendix*, Fig. S13). It should be noted that these results are not clinically significant, and further work has to be done before SimCells can be realistically applied. However, the results in this study have demonstrated that SimCells were able to act as biocatalysts to produce catechol which was cytotoxic against cancer cells but did not kill noncancerous cells. SimCells have some advantages over traditional bacterial therapy in medicine because they are inducible, functional, and controllable (nonreplicating minimal cells).

## Discussion

In this work, we constructed and characterized a different type of minimal cell called SimCells or simple cells, with the intention of applying it as a chassis for reprogramming microorganisms. We developed a universal method to generate chromosome-free SimCells from almost all bacterial genera. These SimCells have the potential to host a minimal genome as a core genetic operating system that can be manipulated in a predictive manner and execute predefined functions (58). Additionally, as SimCells are unable to replicate due to the absence of the chromosome, this alleviates some of the biosafety concerns associated with genetically modified microorganisms (59).

SimCells were readily generated by removal of the chromosome by exploiting the combined activity of I-CeuI endonuclease-mediated DSBs and RecBCD-mediated DNA degradation (Movies S1*A*, S2, and S3). This SimCell generation method is simple and valuable because it can be applicable to bacteria other than *E. coli* (such as *P. putida* and *R. eutropha* in this study) as the I-CeuI recognition sequence is present in 23S rRNA and, thus, occurs several times in the bacterial genome. Studies usually observe chromosomal degradation in *recA* mutants as RecA plays a vital role in DSB repair (15, 28, 60). However, as we generated several DSBs the RecA proteins were probably unable to halt chromosomal degradation by RecBCD.

Unlike protocells that are liposomes or vesicles, the SimCell retains its original bacterial cell membrane. The bacterial membrane houses many vital functions: sensing of environmental signals, generation of ATP via proton gradients created by the electron transport chain, the import of nutrients into the cell, and the export of waste out of the cell (61). To maintain the function and extend the shelf life of SimCells, we have identified the following key requirements: 1) a system to regenerate ATP and the electron carrier, e.g., NAD(P)H, thereby ensuring the maintenance of energy flux and redox reactions; 2) the synthetic design of a physically robust cell structure and stable cellular machinery; 3) machinery repair of central dogma components including DNA, RNA, RNA polymerase, sigma factors, ribosomes, and tRNA; 4) a supply (including recycling) of essential building blocks, such as 12 precursor metabolites, amino acids, and nucleoside triphosphate (NTPs); and 5) continuous removal of reaction by-products and degradation of some RNA and proteins.

Our results support the idea that certain metabolic pathways and machineries (e.g., transcription and translation) are preferentially up-regulated to ensure cell survival and function, which provide insights into the design and construction of minimal cells. We showed that when instructed by plasmid DNA, SimCells are able to produce protein, and this activity was improved after the glycolysis pathway was restored in cells, with about 56% of SimCells being metabolically active. The SimCells in this study sustained functionality for about 10 d until they expended their energy currency. Proteorhopsin (PR) is a light-activated proton pump, which can promote proton motive force and enhance ATP synthesis in the presence of light. It has been found that *E. coli* expressing PR can survive better even in the dark, compared with *E. coli* without PR (62). *E. coli* can assemble a high density of PR molecules (187,000 per cell) on the membrane, which presumably act as a scaffold stabilizing the cytoplasmic membrane, maintaining cellular levels of DNA and RNA and avoiding deterioration of the cytoplasmic membrane, a likely basis for extended viability (62). Hence, PRs in SimCells will not only help ATP regeneration under light but also serve as scaffold to maintain the integrity of cells and extend the shelf life of SimCells. It would be valuable to test if shelf life could be improved with alternative temperatures such as −20 °C and to try different cryoprotectants such as DMSO (dimethyl sulfoxide). Any further design should include genes encoding for a cellular machinery repairing system and pathways for the supply of central metabolites. It is likely that the continuous removal of products and waste could help maintain the equilibrium of SimCells and significantly extend the functional time of this artificial system (63).

SimCells could be engineered to specifically target cancer cells to enhance the efficacy of catechol. For example, expression of cancer biomarker such as carcinoembryonic antigen (CEA) on the surface of SimCells (surface display) would enable SimCells to recognize and bind colon cancer cells (64). This combination of SimCell binding to cancer cells and local delivery of a high concentration of catechol (from aspirin/salicylate) should result in an enhanced killing effect. Additionally, SimCells carrying different regulation systems, such as the recA regulation system (activated by γ-radiation therapy for cancer), could be explored to control the safe release of anticancer drugs generated by SimCells in order to enhance the anticancer effects.

For real-world applications, it is important to effectively remove parent cells and obtain highly purified SimCells. SimCells have much less DNA than parent cells due to the absence of chromosomes, a potentially useful distinguishing feature. Single-cell Raman spectra (SCRS) are label-free biochemical profiles of single cells (65). Preliminary experiments showed that the characteristic DNA bands in SCRS of SimCells were dramatically lower than that of parent cells (*SI Appendix*, Fig. S15). Hence, the Raman bands for DNA (785 and 1,575 cm^−1^) could be used as the sorting criteria for the removal of parent cells using Raman activated cell sorting (RACS) (66). Cells sorted by RACS remain functional (67, 68).

The chromosome-free SimCell will be a universal platform for reprogramming bacterial cells and can drive advancements in cell design and the construction of synthetic cells. SimCells have the potential to open up new frontiers in synthetic biology: they can be utilized as minimal cells to study the basic requirements for life or they can be chassis cells, enabling the development of new and smart systems for biomanufacturing, healthcare, agriculture, and environment.

## Materials and Methods

### Construction of an Endonuclease Expression Plasmid.

The strain*s* and plasmids used in this study are listed in *SI Appendix*, Table S1. Primers used in this study are listed in *SI Appendix*, Table S2. The construction of other plasmids used in this study is described in *SI Appendix*.

The *I-CeuI* endonuclease gene was synthesized by ThermoFisher Ltd (GeneArt) to yield pGeneArt-ICeuI (*SI Appendix*, Fig. S1*A*). In pGeneArt-ICeuI, an extra 15 bp in the pBAD promoter region (69) was added to form a hairpin loop with the corresponding reverse complementary sequence on the *I-CeuI* gene, effectively blocking gene expression and allowing synthesis. The *I-CeuI* gene was subsequently cloned into the pJKR-H-TetR plasmid, a gift from George Church (Addgene plasmid no. 62561) (23) to yield the chromosomal degradation plasmid pJKR-HTetR-ICeuI (*SI Appendix*, Fig. S1*B*).

### Culture Conditions for SimCells.

For SimCell generation, strains containing the pJKR-HTetR-ICeuI plasmid (*SI Appendix*, Fig. S1*B*) were induced with 100 nM anhydrotetracycline (ATc), while strains with pRH121 were induced with 1 µM crystal violet. For SimCell verification, chromosomal expression of GFP was induced with 0.2% arabinose. For protein production, strains containing the pJKR-O-mphR plasmid (or a variation) were induced with 200 μM erythromycin. For SimCell purification, a protocol was adapted from Heinemann and Ankenbauer (33) using D-cycloserine to destroy parent cells.

### Characterization of SimCell Populations.

Flow cytometry analysis was done with an S3e Cell Sorter (Bio-Rad). The FL1 filter was used to detect fluorescence from GFP, which has an excitation/emission at wavelengths 488/507 nm. The FL3 filter was used to detect fluorescence from mCherry, which has an excitation/emission at 587/610 nm.

### Imaging.

A Nikon Fluorescent Microscope Ti Eclipse (Nikon Ltd) was used to take images and videos of bacterial cells. For videos, frames were taken every 15 min for about 24 h. Cells were first stained with 100 µM DAPI and then fixed on an agar slide. The agar contained inducers for *I-CeuI* expression and protein expression when appropriate.

### Longevity of SimCells.

Purified SimCell cultures expressing luminescence (pJKR-OmphR-ilux) or an unstable mCherry variant (pJKR-OmphR-ASV) were kept in 50 mL Falcon tubes shaking at 100 rpm at 37 °C. Every 2 d, 25 µg/mL D-cycloserine was added to the cultures to maintain SimCell purity. At days 1, 3, 5, 10, 14, and 28, aliquots (200 µL, *n* = 4) were taken and measured for luminescence or fluorescence production over 24 h. The maximum reading during this period was recorded. OD600 was compared at t = 0 and t = 24 h to see if there was an increase and therefore contamination by parent cells.

### Proteomics and LC-MS/MS Analysis.

Proteomics analysis was performed as described previously (4). The proteomics data are available on MassIVE with DOI 10.25345/C5HD3F.

### Catechol Quantification Using LC.

Samples were analyzed using LC (Agilent 1120 Compact). The metabolite separation was achieved using a ZORBAZ Eclipse Plus C18 packed with 95 Å pore, 5 µm particle size, and 4.5 × 150 mm column (Agilent). Elution was performed as previously described by Sawyer and Kumar (70). For culture, 300 µL purified SimCells or parent cells in LB were induced or not induced for catechol production in 1.5-mL Eppendorf tubes. Tubes were placed in a shaking incubator overnight at 37 °C. Parent cells and SimCells were spun down at 10,000 × *g*, and the supernatant was analyzed for catechol concentration. OD600 was recorded to calculate the number of cells per mL and subsequently the moles of catechol produced per cell.

### Cell Viability Assay.

Mammalian cells were seeded at a density of 1 × 10^4^ cells per well in 96-well tissue culture-treated plates and incubated for 24 h (*n* = 6). Supplements (catechol, SA, 25 µg/mL D-cycloserine, 10^5^ SimCells) were then added, and cells were incubated for another 24 h. In the case of SimCells, the inducer SA (or PBS) and D-cyloserine were added at the same time to the mammalian cells. Cells were washed twice with PBS then fixed with 1% glutaraldehyde for 30 min. Cells were then stained with 0.5% crystal violet solution for 1 h, washed, and resolubilized with 1% SDS and 10% acetic acid. Absorbance was measured at 595 nm.

### Modeling Energy Consumption in SimCells with the Glycolysis Pathway.

In SimCells which contain the glycolysis pathway, the overall energy E (molar) in the form of ATP and NADH is used to drive protein production. The glycolysis pathway (from glucose to pyruvate) is composed of ∼10 enzymatic reactions operating in series. For simplicity, we considered the concentration of a representative glycolytic enzyme, X. Its conservation can be modeled by the following ordinary differential equation:

$$\frac{dX}{dt}=r_{x1}\frac{E}{E+K_{e,x1}}-D_{x1}X.$$ [1]

The rate of glycolytic enzyme synthesis, $r_{x1}$, has been proposed to hold a simple Michaelis–Menten dependence on the intracellular energy level E. The kinetic constant is $K_{e,x1}$. The rate of enzyme degradation is $D_{x1}$.

The change in the intracellular energy level (in forms of ATP and NADH) is dictated by the energy production from glycolysis, energy consumption for the transcription and translation of glycolytic enzymes, and energy consumption for luminescence:

$$\frac{dE}{dt}=r_{e1}X-a_{x1}r_{x1}\frac{E}{E+K_{e,x1}}-bE.$$ [2]

The rate of energy production via glycolysis is $r_{e1}$. The energy demand per unit rate of glycolytic enzyme synthesis is denoted by $a_{x1}$. The rate of energy consumption for luminescence is b. In this system, the substrate can deplete over time, which could become a limiting factor for energy biogenesis. Therefore, we link the rate of energy production $\left(r_{e1}\right)$ with the substrate concentration S through the Michaelis–Menten kinetics:

$$r_{e1}=r_{e,max}\frac{S}{S+K_{s}}.$$ [3]

The maximum rate of energy production is $r_{e,max}$. The kinetic constant is $K_{s}$. Subsequently, we introduce the dynamic change in the limiting substrate:

$$\frac{dS}{dt}=-\varepsilon r_{e1}X.$$ [4]

The rate of substrate consumption is considered proportional to the rate of energy production through a yield factor ε.

Eqs. **1**–**4** are adopted as the key mechanisms underpinning the observed variation in bioluminescence for a single cell system.

Please see *SI Appendix* for more detailed materials and methods.

### Data Availability Statement.

The proteomics data are available on MassIVE with DOI 10.25345/C5HD3F.

## Supplementary Material

Supplementary File

Supplementary File

Supplementary File

Supplementary File

Supplementary File

Supplementary File

Supplementary File
