# Supplementary material for: Chromosome-free bacterial cells are safe and programmable platforms for synthetic biology
Source: Proc Natl Acad Sci U S A. 2020 Mar 6;117(12):6752–61. doi: 10.1073/pnas.1918859117 (PMC7104398; doi:10.1073/pnas.1918859117)
Supplement: Supplementary File [file pnas.1918859117.sapp.pdf]

Chromosome-free bacterial cells are safe and programmable platforms for synthetic biology

Supplementary Materials

Materials and Methods

Bacterial strains and culture conditions for SimCells

The strains and plasmids used in this study are listed in Table S1. The *E.coli* strain with a GFP expression circuit on the chromosome was a gift from Barry Wanner (CGSC BW31003) [1]. Cultures were grown in LB media at 37°C. When appropriate, 25 μg/mL chloramphenicol, 50 μg/mL kanamycin, 50 μg/mL carbenicillin, 100 μg/mL spectinomycin, or 12.5 μg/mL tetracycline was added to maintain selection pressure.

For SimCell generation, strains containing the pJKR-H-TetR-ICeuI plasmid (Fig. S1b) were induced with 100 nM anhydrotetracycline (ATc). For SimCell verification, chromosomal expression of GFP was induced with 0.2% arabinose. For protein production, strains containing the pJKR-O-mphR plasmid (or a variation) was induced with 200 μM erythromycin. The original pJKR-O-mphR plasmid (Fig. S1d) was from George Church lab (Addgene plasmid #62570) [2].

The glycolysis pathway was induced with 1 mM isopropyl β-D-1-thiogalactopyranoside (IPTG) and supplemented with 10 mM glucose. However, this was not done in the primary experiments shown in the main text as over-production of glycolytic proteins negatively affected cell performance (Fig. S8). It seemed the leaky expression of glycolytic proteins was the ideal balance of providing energy generation without excessive burden on translation and transcription. The leaky behavior of the lac expression system was validated (Fig. S9).

Purification of SimCells

A protocol for minimal cell purification from Heinemann and Ankenbauer was adapted for SimCell purification [3]. Mixed cultures of SimCells and parent cells were treated with D-cycloserine to kill actively dividing cells. D-cycloserine was added at a concentration of 200 μg/mL and cells were left to incubate at 37°C and shake for 1.5 hours before D-cycloserine was added again at the same concentration. After 24 hours of treatment with D-cycloserine, cells were spun down and washed twice with phosphate buffered saline (PBS). When pure SimCell cultures were induced for protein expression, 25 μg/mL D-cycloserine was added every two days to prevent parent cell growth but allow SimCells to be functional.

Quantification of viable (parent) cell populations

www.pnas.org/cgi/doi/10.1073/pnas.1918859117

Cultures were normalized to have the same OD_{600}. Dilutions of cultures were spotted (10 µL in triplicate) onto LB agar plates with no antibiotics. Plates were incubated overnight at 37°C and CFU/mL was calculated the following day.

Construction of various gene-circuits and plasmids

Primers used in this study are listed in Table S2.

Construction of endonuclease expression plasmid

The *I-CeuI* endonuclease gene was synthesized by ThermoFisher Ltd (GeneArt) to yield pGeneArt-ICeuI (Fig. S1a). In pGeneArt-ICeuI, an extra 15 bp in the pBAD promoter region [4] was added to form a hairpin loop with the corresponding reverse complementary sequence on the *I-CeuI* gene, effectively blocking gene expression and allowing synthesis. The pJKR-H-TetR plasmid, a gift from George Church (Addgene plasmid #62561) [2] was used as the expression vector for *I-CeuI* endonuclease. The *I-CeuI* endonuclease gene was amplified with PCR using the primer pair ICeuI_SacI.FOR and ICeuI_XbaI.REV, while the vector backbone (native GFP was excluded) of pJKR-H-TetR was amplified with TetR_XbaI.FOR and TetR_SacI.REV. Products were digested with SacI and XbaI and stitched together with T4 DNA ligase (NEB) to construct the chromosomal degradation plasmid pJKR-HTetR-ICeuI (Fig. S1b).

Construction of glycolysis plasmid

The plasmids pSEVA224-GBI and pSEVA224-GBII, kindly provided by Victor de Lorenzo [5], contain the upper and lower catabolic pathways of glycolysis respectively. The lower catabolic block from pSEVA224-GBII was moved into the multi-cloning site (BamHI and HindIII) on pSEVA224-GBI to yield pSEVA224-GB3 (Fig. S1c), and a complete glycolysis pathway.

Construction of ilux plasmid

An improved version of the classic *lux* operon (sevenfold higher brightness) called *ilux*, was taken from pQE-ilux, a gift from Stefan Hell [6]. The insert was amplified with the primer pair ilux_KpnI.FOR and ilux_BamHI.REV. The vector backbone of pJKR-O-mphR (Fig. S1d) was amplified with Xcherry_BamHI.FOR and Xcherry_KpnI.REV to exclude mCherry. The *ilux* insert and pJKR-O-mphR vector were digested with BamHI and KpnI and ligated to produce pJKR-OmphR-ilux (Fig. S1e).

Construction of unstable mCherry

An *ssrA* protease tag with the ASV variant [7] was added to the C-terminus end of the mCherry sequence in the pJKR-O-mphR plasmid. The entire pJKR-O-mphR plasmid was amplified with

the primers tag.FOR and ASV.REV, which introduces the ASV *ssrA* tag. This pJKR-OmphR-ASV plasmid (Fig. S1f) now expresses an unstable version of mCherry called mCherry-ASV.

Construction and application of broad host-range SimCell-generating plasmids

To demonstrate SimCell formation in a wider array of organisms we constructed the plasmid pRH121 (Fig. S5a) via a HiFi Assembly approach, based on the pSEVA231 backbone kindly provided to us by Victor de Lorenzo [5]. Primers for the amplification of the various parts of pRH121 are displayed in Table S2. Tight transcriptional control of *I-CeuI* was deemed mandatory to avoid the cellular defences of various bacteria that may inactivate the SimCell-forming machinery. The Jungle Express repressor system has been shown to provide exceptionally tight transcriptional control in several different species, including *P. putida* [8]. This tight level of control was also witnessed in *R. eutropha* (Fig. S6). Therefore, in pRH121 the EilR repressor and P_{JExD} promoter from pJC580 (JBEI Part ID: JPUB_010723, Fig. S5b) governed the transcription of an *I-CeuI* gene that had been codon optimised for *R. eutropha* H16 (synthesized by Integrated DNA Technologies).

The final pRH121 plasmid was sequenced and transferred to both *R. eutropha* H16 and *P. putida* UWC1 by conjugation via the S17-1 *E. coli* donor. *R. eutropha* and *P. putida* cells containing pRH121 were then selected on LB plates containing 50 µg/mL kanamycin, and either 50 µg/mL gentamicin or 50 µg/mL rifampicin, respectively. I-CeuI expression was induced in both strains with 1 µM crystal violet.

Characterization of SimCell populations

Flow cytometry analysis was done with an S3e Cell Sorter (Bio-Rad). The FL1 filter was used to detect fluorescence from GFP, which has an excitation/emission at wavelengths 488 nm/507 nm. The FL3 filter was used to detect fluorescence from mCherry, which has an excitation/emission at 587/610 nm.

Imaging

Cells were visualized with a Nikon Ti Eclipse. To visually compare SimCell versus parent cell growth, cells were fixed in agar containing 1.2% Noble Agar, 10X diluted LB media, 100 nM anhydrotetracycline (ATc), 0.2% arabinose and 200 µM erythromycin with the thickness of a coverslip on a glass slide. The slide was incubated overnight at room temperature and visualized the following day. To take the videos demonstrating SimCell synthesis of mCherry, cells were stained with 100 µM DAPI and fixed in the same agar with the same components except LB. Visualisation of SimCell formation from *R. eutropha* and *P. putida* containing pRH121 was conducted as described previously, but used 1 µM crystal violet to induce *I-CeuI* expression. Frames were taken every 15 min for about 24 hr.

Longevity of SimCells

Purified SimCell cultures expressing luminescence (pJKR-OmphR-ilux) or an unstable mCherry variant (pJKR-OmphR-ASV) were kept in 50 mL Falcon tubes shaking at 100 rpm at 37°C. Every two days 25 µg/mL D-cycloserine was added to the cultures to maintain SimCell purity. At days 1, 3, 5, 10, 14, and 28, aliquots (200 µL, n=4) were taken and measured for luminescence or fluorescence production over 24 hours. The maximum reading during this period was recorded. OD600 was compared at t=0 and t=24 hours to see if there was an increase and therefore contamination by parent cells.

Sample preparation for proteomics and LC-MS/MS analysis:

The *E. coli* DH5α pellets of the wildtype, with pLO11 and with pLO11-ICeuI, were resolubilized in a solution containing 8 mol/l urea (Merck, Darmstadt, Germany) and 2 mol/l thiourea (Merck, Darmstadt, Germany). Solubilised cells were disrupted by 5 cycles of freezing in liquid nitrogen and subsequent incubation at 30°C for 10 min. Afterwards, medium intensity ultrasonic pulses were applied for 30 seconds. Cell fragments were removed by centrifugation at 20,000 g for 1h at room temperature. The resulting supernatant was collected.

The final protein concentration was estimated by Bradford assay. Protein solutions containing 4 µg protein were incubated overnight with 160 ng Sequencing Grade Modified Trypsin (Promega, Madison, WI, USA). Incubation was stopped after 16 hr by application of acetic acid (Carl Roth, Karlsruhe, Germany) to a final concentration of 1%.

The resulting peptides were purified using C18 PureSpeed LTS tips (Mettler-Toledo, Gießen, Germany) with 20 µl electric multichannel pipette (Mettler-Toledo, Gießen, Germany). After freeze-drying peptides were resolved in Buffer A consisting of 2% acetonitrile (J.T. Baker®, part of Fisher Scientific, Waltham, MA, USA), 0.1% acetic acid (Carl Roth, Karlsruhe, Germany) in water (J.T. Baker®, part of Fisher Scientific, Waltham, MA, USA), and subjected to LC-MS/MS analysis. To perform data independent acquisition (DIA) HRM peptide standard (Biognosys AG, Schlieren, Switzerland) was spiked in.

Data acquisition was done on a Q Exactive mass spectrometer (Thermo-Fisher Scientific, Idstein, Germany) in combination with an UltiMate 3000 RSLC (Thermo-Fisher Scientific, Idstein, Germany) and a Nanospray Flex Ion Source (Thermo-Fisher Scientific, Idstein, Germany). For the construction of the *E. coli* ion library, each sample was analysed in data-dependent acquisition (DDA) as well as in DIA mode.

Raw data analysis and protein identification

Spectronaut Pulsar X (v 12.0.20491.0.25470; Biognosys AG, Schlieren, Switzerland) was used to generate the *E. coli* DH5α ion library. Database was a FASTA file containing 4,288

identical proteins of *E. coli* DH5α (ISNDC; Date 2018/11/09). Digestion rule was trypsin/P with a maximum of 2 allowed missed cleavages. No fixed modifications were set while Oxidation (M) was considered as variable modification. Only the 7-52 AA peptides were preselected and those with more than 6 transitions were finally considered for the library. The final *E. coli* library contained 24,246 peptides and 2,274 proteins, of which 1,228 were found in DIA dataset. The proteomics data has been uploaded to MassIVE with DOI number: 10.25345/C5HD3F.

DIA raw data were analysed with Spectronaut Pulsar X (v 12.0.20491.0.25470; Biognosys AG, Schlieren, Switzerland). The generated raw data were further analysed in a R-environment (v 3.5.1). MS2-peak-areas were median normalized on replicate level and conditions were compared on peptide level using reproducibility-optimized peptide change averaging method (ROPECA)[9]. Candidates with adjusted P-values (Benjamini and Hochberg) < 0.05 and fold changes of +/- 1.5 were considered as significantly changed. Mass spectrometry settings, mass windows for DIA analysis, and R packages used for analysis are listed in a related study [10].

The heatmaps were generated using the seaborn Python data visualization library [11]. For the dendrograms, correlation was used as the distance metric with single-linkage clustering. Protein abundances were row scaled to show the fold change.

Modelling unstable mCherry-ASV production and degradation

The time-dependent change in the mCherry-ASV concentration C is modelled as:

$$\frac{dC}{dt} = r_{\mathrm{c}} \frac{E}{E+K_{e,\mathrm{c}}} - k_d Y \frac{C}{C+K_c} \frac{E}{E+K_{e,cd}} \quad (5)$$

The rate of mCherry-ASV synthesis is r_{c}, which also possesses a Michaelis-Menten dependence on the energy level, with $K_{e,\mathrm{c}}$ as the kinetic constant. The degradation of mCherry-ASV is achieved by the protease Y. Therefore the degradation process is considered to follow the Michaelis-Menten kinetics for enzymatic reactions. The (maximum) rate of mCherry-ASV degradation is k_d. As proteolysis of mCherry-ASV is also energy dependent, substrate level (C) and energy level (E) are both incorporated into the kinetic terms, with K_c and $K_{e,cd}$ being the corresponding kinetic constants, respectively.

The dynamic change in the concentration of protease is modelled as

$$\frac{dY}{dt} = -D_y Y \quad (6)$$

Where D_y is the rate of protease degradation. New proteases are not synthesized during the considered process.

The change in glycolytic enzyme follows a similar form to **Equation (1)** shown in the main text

$$\frac{dX}{dt} = r_{x2}\frac{E}{E+K_{e,x2}} - D_{x2}X \quad (7)$$

Where r_{x2} is the rate of glycolytic enzyme synthesis, $K_{e,x2}$ is the kinetic constant, and D_{x2} is the rate of enzyme degradation.

The change in the overall energy can be calculated by the energy production by glycolysis, energy consumption for the expression of glycolytic enzymes, energy consumption for mCherry-ASV synthesis and that for mCherry-ASV hydrolysis.

$$\frac{dE}{dt} = r_{e2}X - a_{x2}r_{x2}\frac{E}{E+K_{e,x2}} - a_c r_{\mathrm{c}}\frac{E}{E+K_{e,\mathrm{c}}} - a_{cd}k_d Y\frac{C}{C+K_c}\frac{E}{E+K_{e,cd}} \quad (8)$$

Where r_{e2} is the rate of energy production via glycolysis. As the substrate is shown to be irrelevant in this system, r_{e2} is considered as a constant. The parameters a_{x2}, a_c and a_{cd} denote the energy demands per unit rate of glycolytic enzyme synthesis, mCherry synthesis and mCherry degradation, respectively.

Finally, the wearing-out of cell machinery is modelled by the time-dependent decay of protein synthesis rate, applied to both glycolytic enzymes and mCherry-ASV:

$$\frac{dr_c}{dt} = -w \ (9)$$

$$\frac{dr_{x2}}{dt} = -w \ (10)$$

Where w is the rate of wearing-out of cell machinery.

Equations (5)-(10) were used to describe the potential mechanisms that govern the experimental observation of the mCherry-ASV production.

Model simulation

Equations (1)-(4) and **Equations (5)-(10)** were solved via ODE solver ode45 in MATLAB 2016a. For modelling energy consumption in SimCells with the glycolysis pathway, the initial condition was set to $X_0 = 0.3$, $S_0 = 12$ and $E_0 = 0$. For modelling unstable mCherry-ASV production and degradation, the initial condition was set to $C_0 = 0$, $Y_0 = 0.01$, $X_0 = 0$, $E_0 = 0.4$, $r_{c0} = 0.1$ and $r_{x20} = 0, 0.02, 3$ to correspond to different expression levels of glycolytic enzymes.

Catechol quantification using liquid chromatography (LC)

Samples were analysed using liquid chromatography (Agilent 1120 Compact, California, US). The metabolite separation was achieved using a ZORBAZ Eclipse Plus C18 packed with 95

Å pore, 5 µm particle size and 4.5 × 150 mm column (Agilent, US). Elution was performed using isocratic mixture of water, methanol and acetic acid (690:280:30) as previously described by Sawyer and Kumar at 0.5 ml min-1 for 10 min [12]. The oven temperature was 30°C. The injection volume was 5 µl. The UV detector was set to a wavelength of 275 nm for catechol detection. Data were collected at an acquisition rate of 5Hz. Control, experimental samples and catechol standards were run sequentially for comparison (n=3). For culture, 300 µL purified SimCells or parent cells in LB were induced or not induced for catechol production in 1.5 mL Eppendorf tubes. Tubes were placed in a shaking incubator overnight at 37°C. Parent cells and SimCells were spun down at 10,000 x *g* and the supernatant was analyzed for catechol concentration. OD600 was recorded to calculate the number of cells/mL and subsequently the moles of catechol produced per cell.

Cell culture

The therapeutic effect of catechol was tested on rhabdomyosarcoma (RD) cancer cell line (ATCC no. CCL-136), glioblastoma (Mo59K) cancer cell lines (ATCC no. CRL-2365), and A549 lung cancer cell line (a gift from Len Seymour at the University of Oxford, UK). A fibroblast control cell line (Fibroblasts) was also used (a gift from Jo Poulton at the University of Oxford, UK). Cells were grown in DMEM (Dulbecco's Modified Eagle Medium) - high glucose supplemented with 10% FBS (fetal bovine serum), 2 mM L-Glutamine, 100 U/mL penicillin, and 0.1 mg/mL streptomycin. Cells were maintained at 37°C in a 5% CO2 incubator and passaged whenever confluent (approximately every 5 days). When SimCells were applied to eukaryotic cells, 25 µg/mL D-cycloserine was also added to maintain SimCell purity.

Cell viability assay

Cells were seeded at a density of 1 x 10^4 cells/well in 96-well tissue culture-treated plates and incubated for 24 hours (n=6). Supplements (catechol, salicylic acid (SA), 25 µg/mL D-cycloserine, 10^5 SimCells) were then added and cells were incubated for another 24 hours. In the case of SimCells, the inducer SA (or PBS) and D-cyloserine were added at the same time to the mammalian cells. Mammalian cells were washed twice with PBS then fixed with 1% glutaraldehyde for 30 min. Cells were then stained with 0.5% crystal violet solution for 1 hour, washed, and resolubilized with 1% SDS (sodium dodecyl sulfate) and 10% acetic acid. Absorbance was measured at 595 nm.

Table S1: A list of strains and plasmids used in this study.

Bacterial strains or plasmids	Genotype, description	Reference or source
**Strains**		
Escherichia coli K12 BW31003	F-, Δ(araD-araB)567, ΔlacZ4787(::rrnB-3), λ-, att80(CmR)::KZ28araBp-GFP, Δ(rhaD-rhaB)568, hsdR514	Coli Genetic Stock Center (CGSC) [1]
ICeuI+ Glycolysis+	*E. coli:* pJKR-O-mphR + pJKR-HTetR-ICeuI + pSEVA224-GB3	This study
ICeuI+ Glycolysis-	*E. coli:* pJKR-O-mphR + pJKR-HTetR-ICeuI + pSEVA224	This study
ICeuI- Glycolysis+	*E. coli:* pJKR-O-mphR + pJKR-H-TetR + pSEVA224-GB3	This study
ICeuI- Glycolysis-	*E. coli:* pJKR-O-mphR + pJKR-H-TetR + pSEVA224-GB3	This study
R. eutropha H16	Wildtype strain, Gen^R (also *Cupriavidus necator* H16)	A gift from Oliver Lenz at Technische Universitaet Berlin
H16ICeuI	*R. eutropha* H16: pRH121	This study
P. putida UWC1	Rif^R variant of *P. putida* KT2440	Lab collection
UWC1 ICeuI	*P. putida* UWC1: pRH121	This study
**Plasmids**		
pJKR-H-TetR	Plasmid with GFP production controlled by TetR, high copy number, Cb^R	[2]
pJKR-HTetR-ICeuI	Plasmid with I-CeuI endonuclease controlled by TetR, high copy number, Cb^R	This study
pJKR-O-mphR	Plasmid with mCherry production controlled by MphR, medium copy number, $Spec^R$	[2]
pSEVA224	Empty vector on which the other glycolysis plasmids on based on, Kan^R	[5]
pSEVA224-GBI	Plasmid with upper glycolysis catabolic pathway (*glk, pgi, pfkA, fbaA, tpiA*), Kan^R	[5]
pSEVA224-GBII	Plasmid with lower glycolysis catabolic pathway (*gapA, pgk, gpmA, eno, pykF*), Kan^R	[5]
pSEVA224-GB3	Plasmid with entire glycolysis pathway, low copy number, Kan^R	This study

pQE-ilux	Plasmid with ilux under constitutive expression, Kan^R	[13]
pJKR-OmphR-ilux	Plasmid with ilux production controlled by MphR, medium copy number, $Spec^R$	This study
pJKR-OmphR-ASV	Plasmid with an ASV *ssrA* tag at the end of mCherry, half-life mCherry production is controlled by MphR, medium copy number, $Spec^R$	This study
pSEVA231	Empty vector backbone with broad host-range origin of replication (pBBR1), origin of transfer (oriT), Kan^R	[14]
pJC580	Source of P_{JExD} Jungle Express promoter and EilR transcriptional repressor, ColE1, RK2, Kan^R	Joint Bioenergy Institute (JBEI) and [15]
pLO11	Source of *araC* and P_{araBAD}, RK2, Tc^R	A gift from Oliver Lenz at Technische Universitaet Berlin
pRH121	pSEVA231 backbone with ICeuI endonuclease (codon-optimised for *R. eutropha* H16) and *araC* under the control of P_{JExD}, BBa_B0024 transcriptional terminator, *mrfp1* under the control of P_{araBAD}	This study
pSalAR-gfp-full	pGEM-T backbone with *salA, gfp,* and *salR* under control of P_{sal}, Tc^R	Lab collection [16]
pSalA_Km_xylR	Defective version of *salA* due to an insertion of a kanamycin resistance marker	Lab collection [17]

Table S2: Primers used in this study.

Oligo name	Oligo sequence (5'->3')	Part
**ICeuI_SacI.FOR**	CATTAGGAGCTCATGTCAAACTTTATACTTAAACC	*I-CeuI*
**ICeuI_XbaI.REV**	CATTAGTCTAGACTACTTTATACCTTTTTTAT	
**TetR_XbaI.FOR**	CATTAGTCTAGACATAACCCTAATGAGTGAGC	pJKR-H-TetR backbone
**TetR_SacI.REV**	CATTAGGAGCTCATGTATATCTCCTTCTTAAAG	
**ilux_KpnI.FOR**	CATTAGGGTACCATGACTAAAAAAATTTCATTC	*ilux* operon
**ilux_BamHI.REV**	CATTAGGGATCCGCTAGCTTGGATTCTCACC	
**Xcherry_BamHI.FOR**	CATTAGGGATCCCTAATGAGTGAGCTAACTTAC	pJKR-O-mphR backbone
**Xcherry_KpnI.REV**	CATTAGGGTACCATGTATATCTCCTTCTTAAAG	
**tag.FOR**	TTAATAAGCTTGAGAGCTAATGAGTGAGCTAACTTACATTAATTGCGT	ASV *ssrA* tag
**ASV.REV**	CTCTCAAGCTTATTAAACTGATGCAGCGTAGTTTTCGTCGTTTGCTGCGGATCCCTTGTACAGCTCGTCCATGCCGCCGG	
**Construction of pRH121 via HiFi Assembly**		
**oligoRH1211_for**	CTCCTGTGTGAAATTGTTATCCGC	pSEVA231 backbone
**oligoRH1211_rev**	ACCTGCAGGCATGCAAGC	
**oligoRH1212_for**	AAGCTTGCATGCCTGCAGGTGACGTCTTACGAAAATAACTC	EilR-P_{JExD}
**oligoRH1212_rev**	TAAAGTTAAAAGATCTTTTGAATTCAAAGTTG	
**oligoRH1213_for**	CAAAAGATCTTTTAACTTTAAGAAGGAGATATACATG	*I-CeuI*
**oligoRH1213_rev**	CTAGTAGGTTTCCTGTGTGAACTACTTTATACCTTTTTTATAATTACGAG	
**oligoRH1214_for**	TATAAAGTAGTTCACACAGGAAACCTACTAGATGGCTGAAGCGCAAAATG	*araC*
**oligoRH1214_rev**	TTTATTATTTTTATGACAACTTGACGGC	
**oligoRH1215_for**	AGTTGTCATAAAAATAATAAAAAAGCCGGATTAATAATCTG	BBa_B0024 terminator
**oligoRH1215_rev**	TTGGTTTCTTTCACACTGGCTCACCTTC	
**oligoRH1216_for**	GCCAGTGTGAAAGAAACCAATTGTCCATATTG	P_{araBAD} promoter
**oligoRH1216_rev**	CCTTCTTAAAATGGAGAAACAGTAGAGAG	
**oligoRH1217_for**	GTTTCTCCATTTTAAGAAGGAGATATACATATGGCG	*mrfp1*
**oligoRH1217_rev**	ATAACAATTTCACACAGGAGCCTAGGATGGGCCCTTAAGCACCGGTGGAGTG	

Table S3: Chromosomal degradation rates of *E. coli, P. putida, and R. eutropha*.

**Strain**	**Genome size (Mbp)**	**No. of restriction sites**	**Total degradation time (min)**	**Degradation rate (bp/sec)**
E. coli I-CeuI+	4.6	7	645	200
P. putida I-CeuI+	6.1	6	450	225
R. eutropha I-CeuI+	7.4	3	840	147

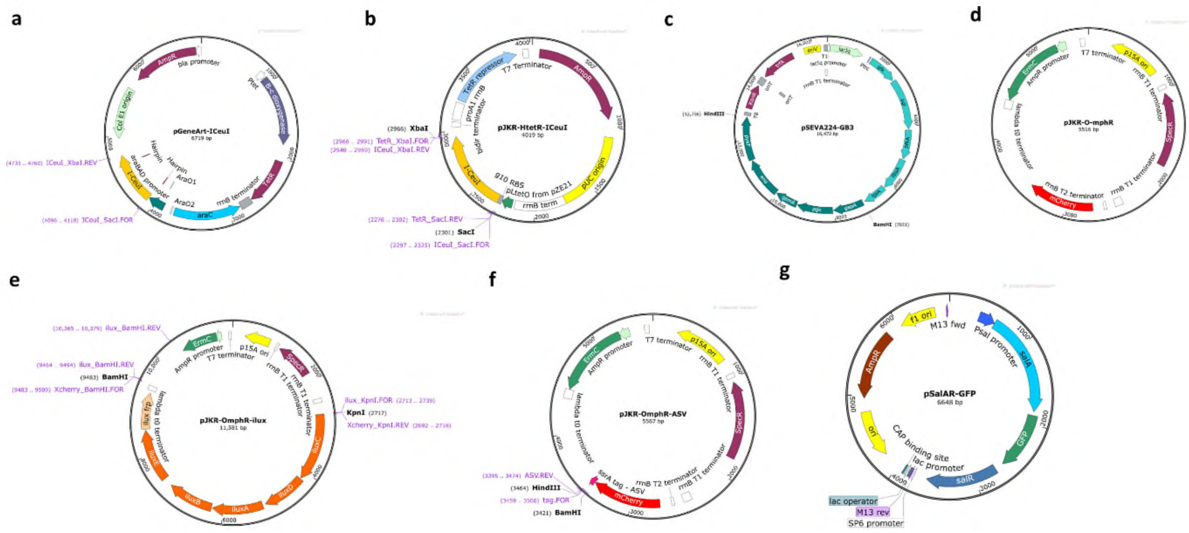

Figure S1: SnapGene maps of plasmids used in this study. **a,** pGeneArt-ICeuI **b,** pJKR-HTetR-ICeuI **c,** pSEVA224-GB3 **d,** pJKR-O-mphR **e,** pJKR-OmphR-ilux **f,** pJKR-OmphR-ASV **g,** pSalAR-GFP

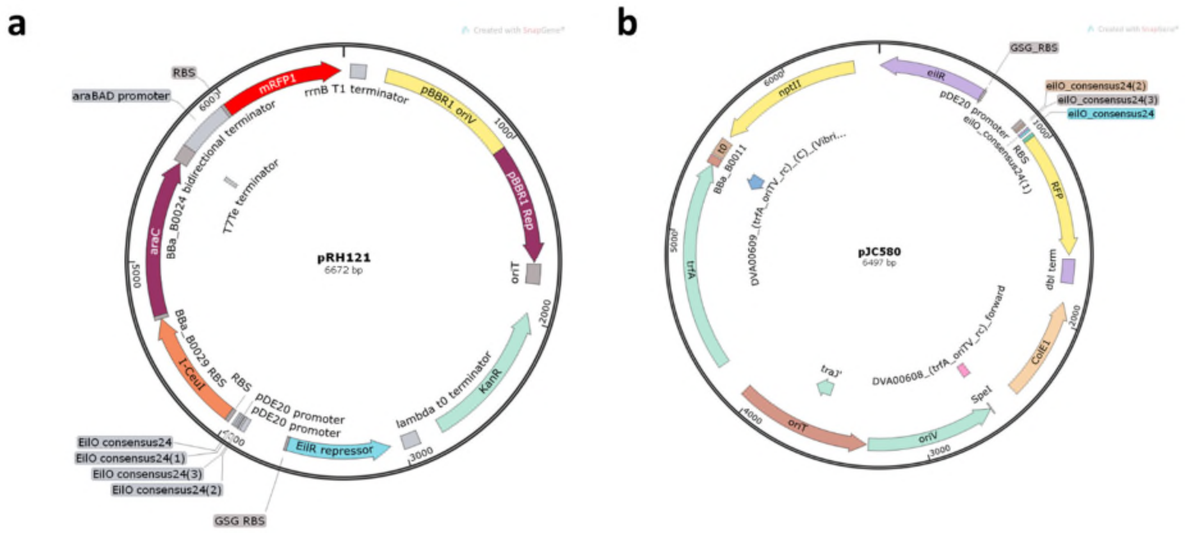

Figure S2: SnapGene maps of broad host-range SimCell-generating plasmids. **a,** pRH121 **b,** pJC580.

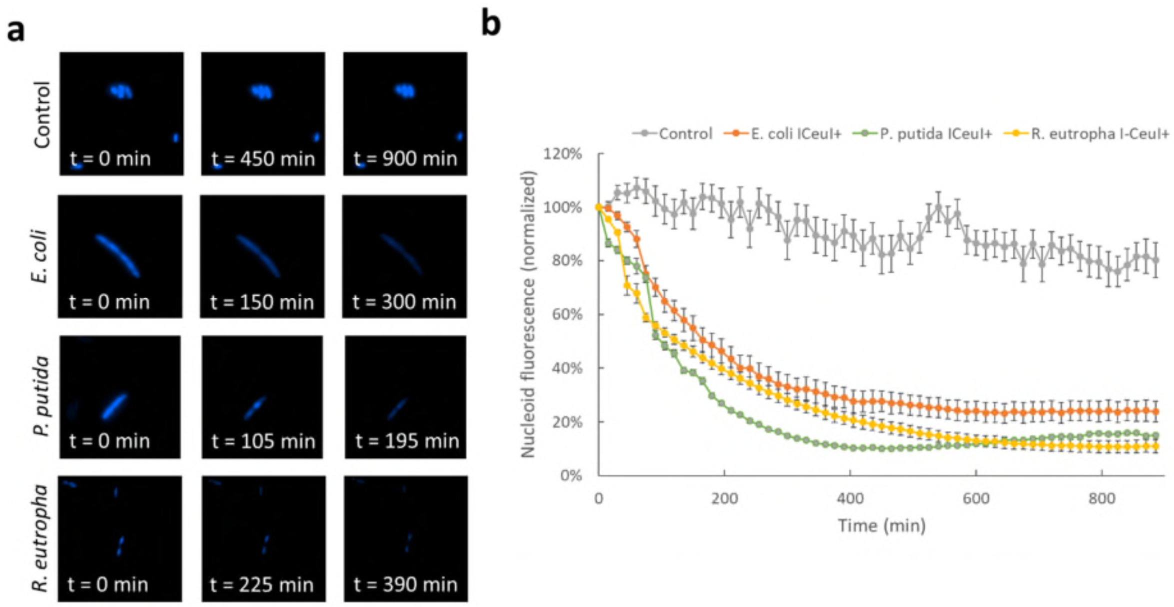

Figure S3: The fluorescence intensity of DAPI is lost over time after *I-CeuI* induction. The nucleoid DNA of strains were stained with DAPI. The *E. coli*, *P. putida*, and *R. eutropha* strains contain a plasmid with *I-CeuI,* while *E. coli* without *I-CeuI* acted as the control. **(a)** Screenshots of the time-lapse videos show the gradual decrease in fluorescence intensity of I-CeuI+ strains but not in the control (I-CeuI-). **(b)** Normalized fluorescence intensity of cells over time. Strains with I-CeuI+ show a rapid loss of DAPI while what little variation exhibited by the control (I-CeuI-) is likely due to noise. Data show means ± SE, n=10.

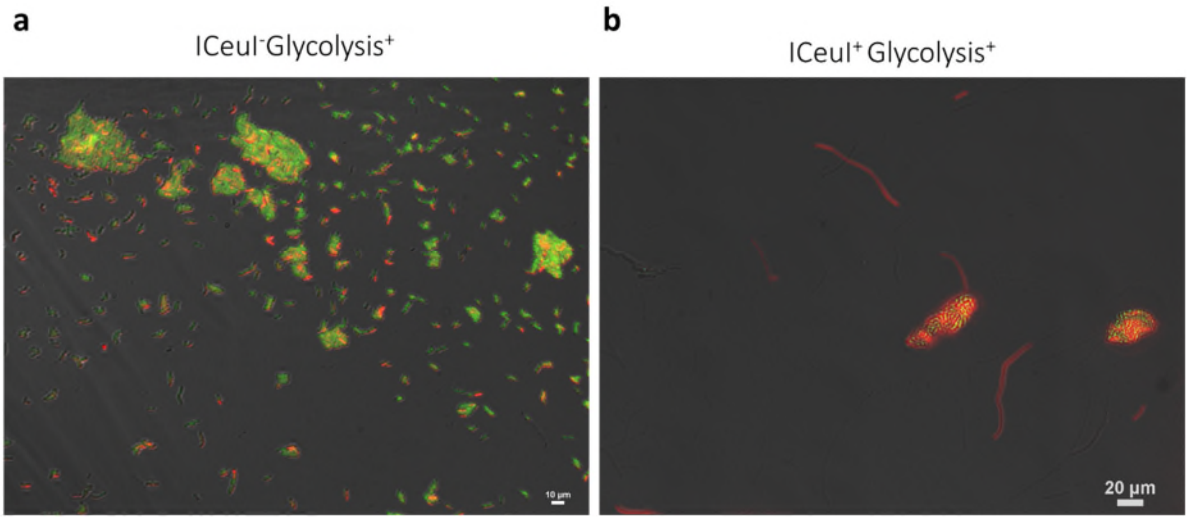

Figure S4: I-CeuI+ and ICeuI- strains with the glycolysis pathway grown overnight in agar. **(a)** In a culture with only normal cells (no *I-CeuI*), they readily formed colonies and produced both GFP and mCherry. **(b)** In an I-CeuI+ strain, a few remaining parent cells formed colonies and produced both GFP and mCherry, while the majority of cells did not form colonies, indicating that they were SimCells (non-replicative) that expressed mCherry and not GFP (chromosome-free).

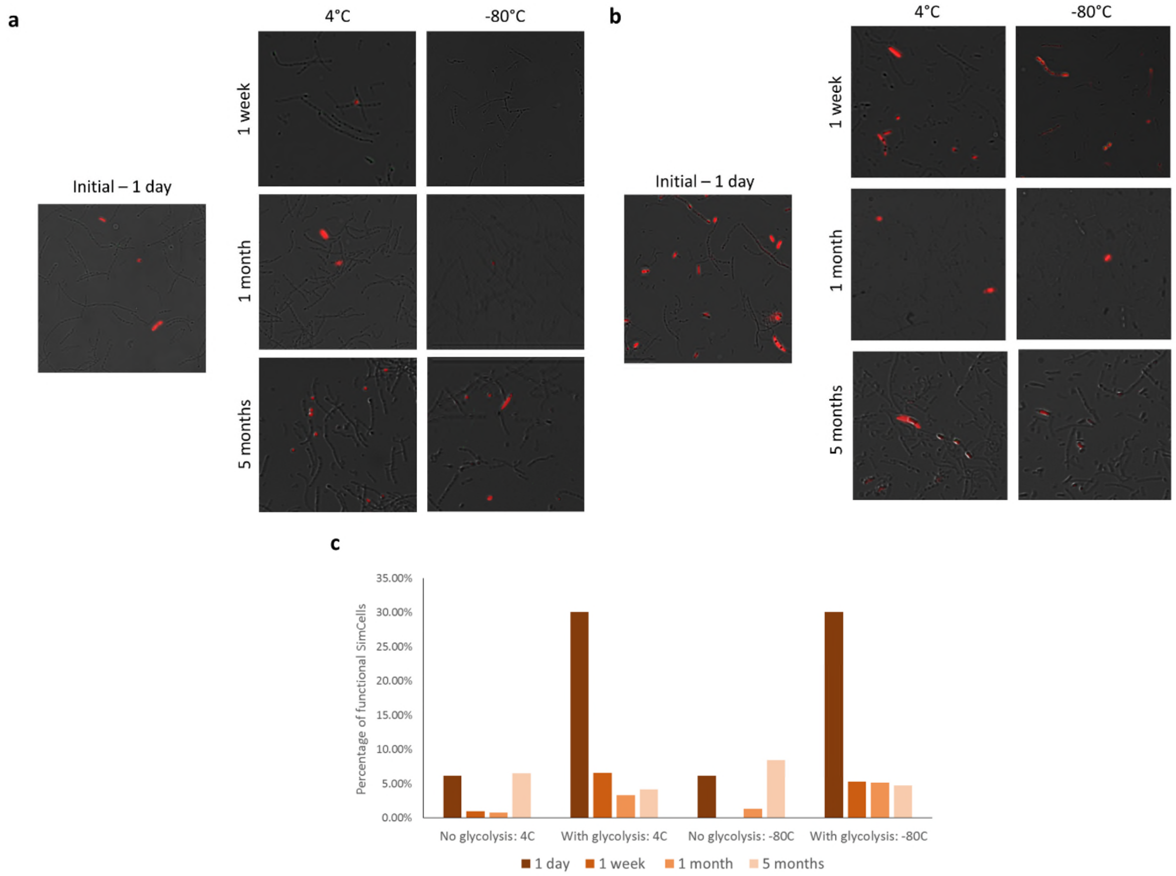

Figure S5: Shelf-life of purified SimCells stored at 4°C and -80°C. Their functionality was tested after 1 day, 1 week, 1 month, and 5 months in storage. Storage at 4°C seemed to have preserved metabolic activity of SimCells **(a)** without glycolysis or **(b)** with glycolysis, better than storage at -80°C. **(c)** Estimation of the proportion of functional SimCells based on microcopy images. Storage severely affected the number of SimCells that could produce mCherry.

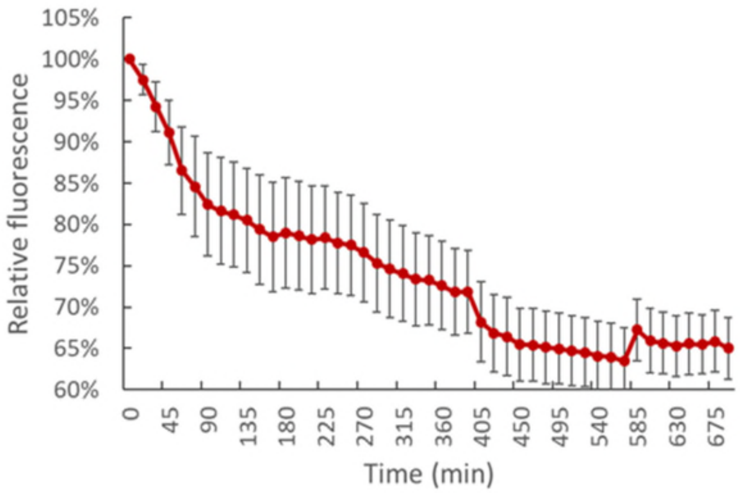

Figure S6: Fluorescence produced by unstable mCherry with the ASV variant of the *ssrA* protease tag. After addition of this tag, mCherry has a half-life time of about 96 min.

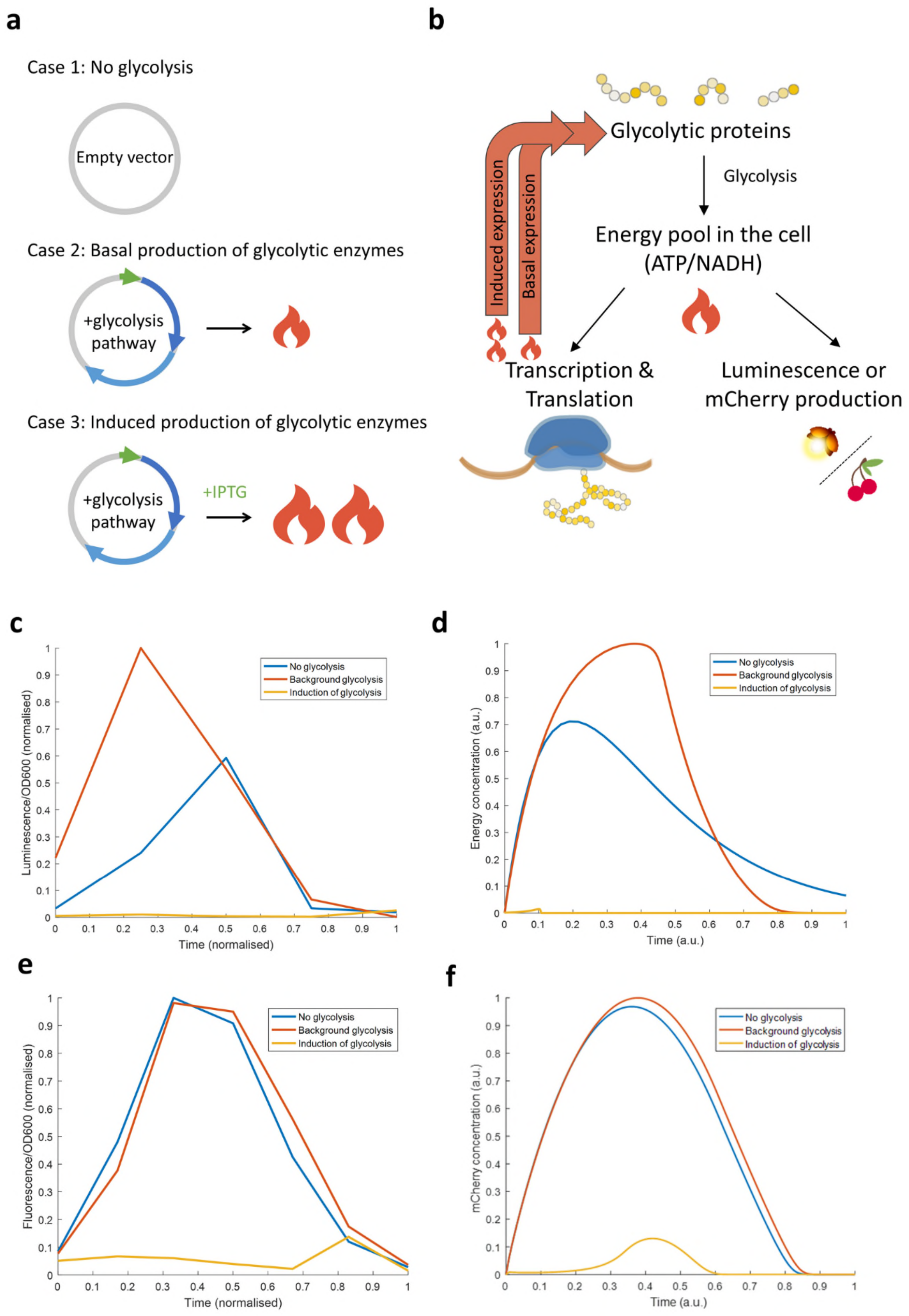
a
Case 1: No glycolysis
Empty vector
Case 2: Basal production of glycolytic enzymes
+glycolysis pathway
Case 3: Induced production of glycolytic enzymes
+glycolysis pathway
+IPTG
b
Glycolytic proteins
Induced expression
Basal expression
Glycolysis
Energy pool in the cell (ATP/NADH)
Transcription & Translation
Luminescence or mCherry production
c
Luminescence/OD600 (normalised)
Time (normalised)
No glycolysis
Background glycolysis
Induction of glycolysis
d
Energy concentration (a.u.)
Time (a.u.)
e
Fluorescence/OD600 (normalised)
Time (normalised)
f
mCherry concentration (a.u.)
Time (a.u.)

Figure S7: Mathematical model of luminescence production in SimCells. **(a-b)** Schematic of experimental setup and concept of the model. The energy available in the cell is affected by the expression of the cargo genes and the transcription and translation machinery. The production of glycolytic proteins, especially when induced with IPTG, places a heavy metabolic load on these machinery. **(c)** Line graph representation of luminescence production data from Fig. 4a**. (d)** Graphical result of the mathematical model closely resembles with the trend of the experimental data for luminescence production over time. When the glycolysis pathway is induced, SimCells are overwhelmed by the additional energy burden so cannot produce luminescence. When glycolytic proteins are present at background levels there is added benefit in terms of better luminescence production when compared to SimCells without the glycolysis pathway. But this benefit eventually wears off after 5 days and the performance of SimCells background glycolysis and no glycolysis are comparable. **(d)** Line graph representation of fluorescence (mCherry-ASV) production data from Fig. 4b**. (f)** Graphical simulation of the model for mCherry-ASV production closely resembles the model. The advantage of the glycolysis pathway is less apparent, likely because *mCherry-ASV* is only one gene compared to the *luxCDABE* operon which consists of five genes. The model predicts induction of glycolysis will briefly yield a small increase in mCherry concentration, which was observed in the experiment. This is because a suitable concentration of glycolytic proteins will eventually be released but the transcription and translation machinery will have been worn out before the benefits of the glycolytic proteins will be realized.

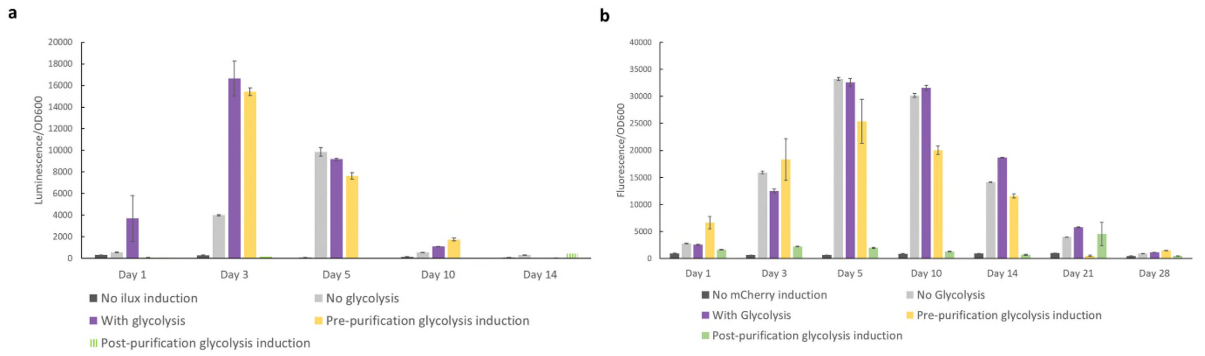

Figure S8: Induction of the glycolysis pathway overwhelmed SimCells. In pre-purification glycolysis induction, IPTG and glucose were added to the overnight cultures which contained a mix of SimCells and parent cells. In post-purification glycolysis induction, IPTG and glucose were added to purified SimCell cultures. **(a)** With pre-purification glycolysis induction, SimCells could produce luminescence for about 10 days, but post-purification induced SimCells could not produce any luminescence. **(b)** With pre-purification glycolysis induction, fluorescence detected from unstable mCherry stopped increasing after 5 days (likely the point where transcription and translation did not have enough energy to be operational). When a pure SimCell culture was made to produce more glycolytic proteins, insignificant amounts of mCherry was produced, as SimCells were overwhelmed by the energy cost of transcription and translation. Data show means ± SE, n=4.

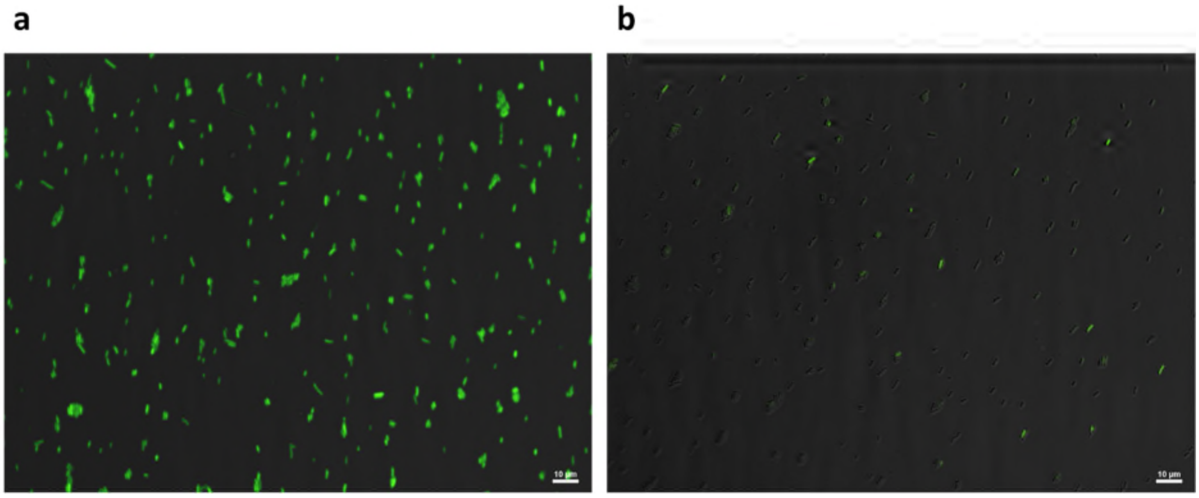

Figure S9: Integrity of the lac reporter system. The strain *E. coli* BW31005 with a chromosomal GFP reporter under control of the lac operon was **(a)** induced and **(b)** not induced with 1mM IPTG for GFP expression. Without any induction, there was leaky expression of GFP due to the weak binding of the LacI repressor to the P_{trc} promoter.

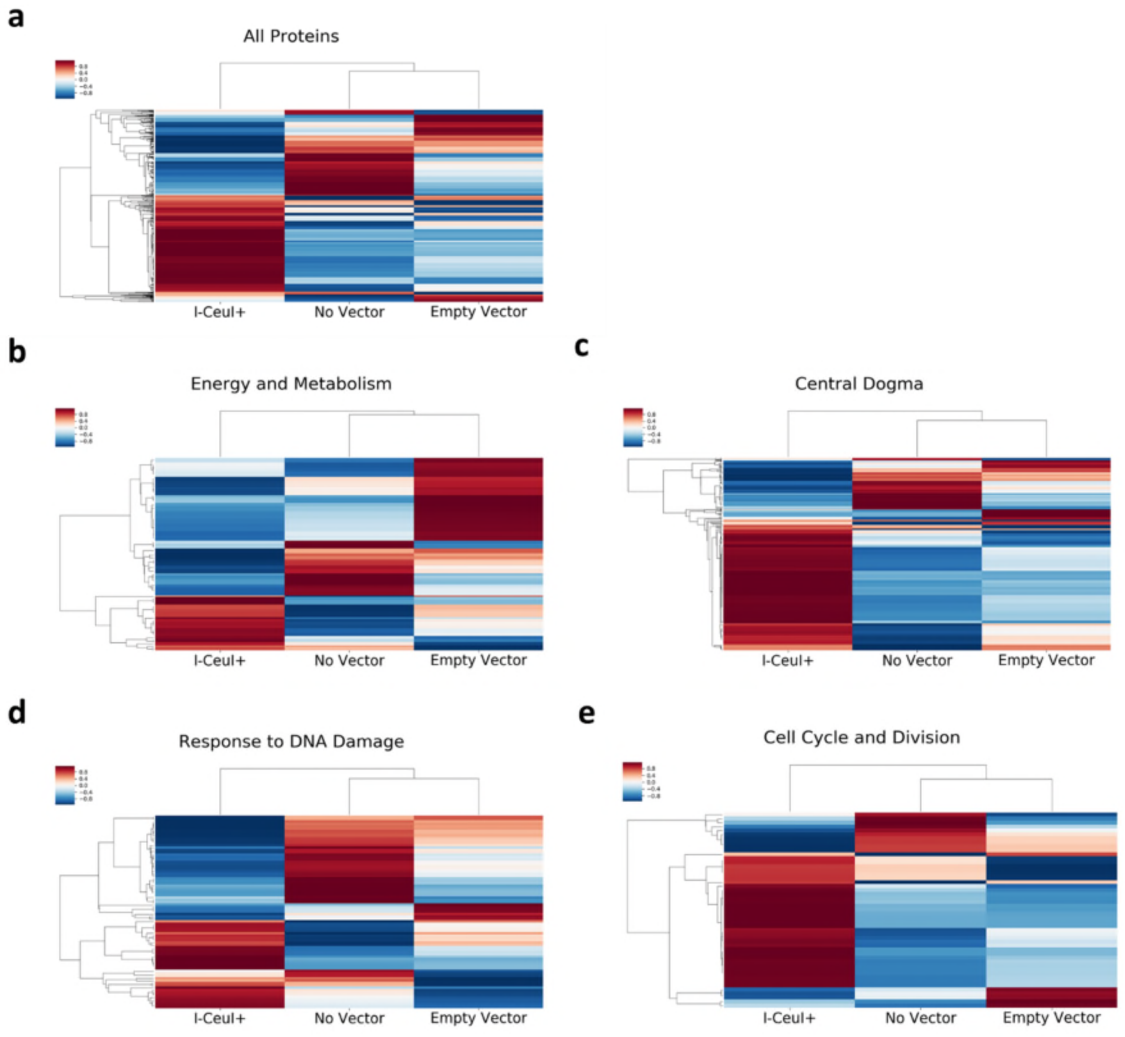

Figure S10: Dendrograms and their associated heatmaps of the relative protein abundance profile of DH5α strains. A cluster analysis was used and the data was row scaled. In the I-CeuI+ strain, the proteome had a distinct abundance profile when compared to the proteomes of the control strains (no vector, empty vector). **(a)** All proteins that were detected and identified. **(b)** Proteins associated with energy and metabolism. **(c)** Proteins associated with central dogma. **(d)** Proteins associated with DNA damage response. **(e)** Proteins associated with cell cycle and division.

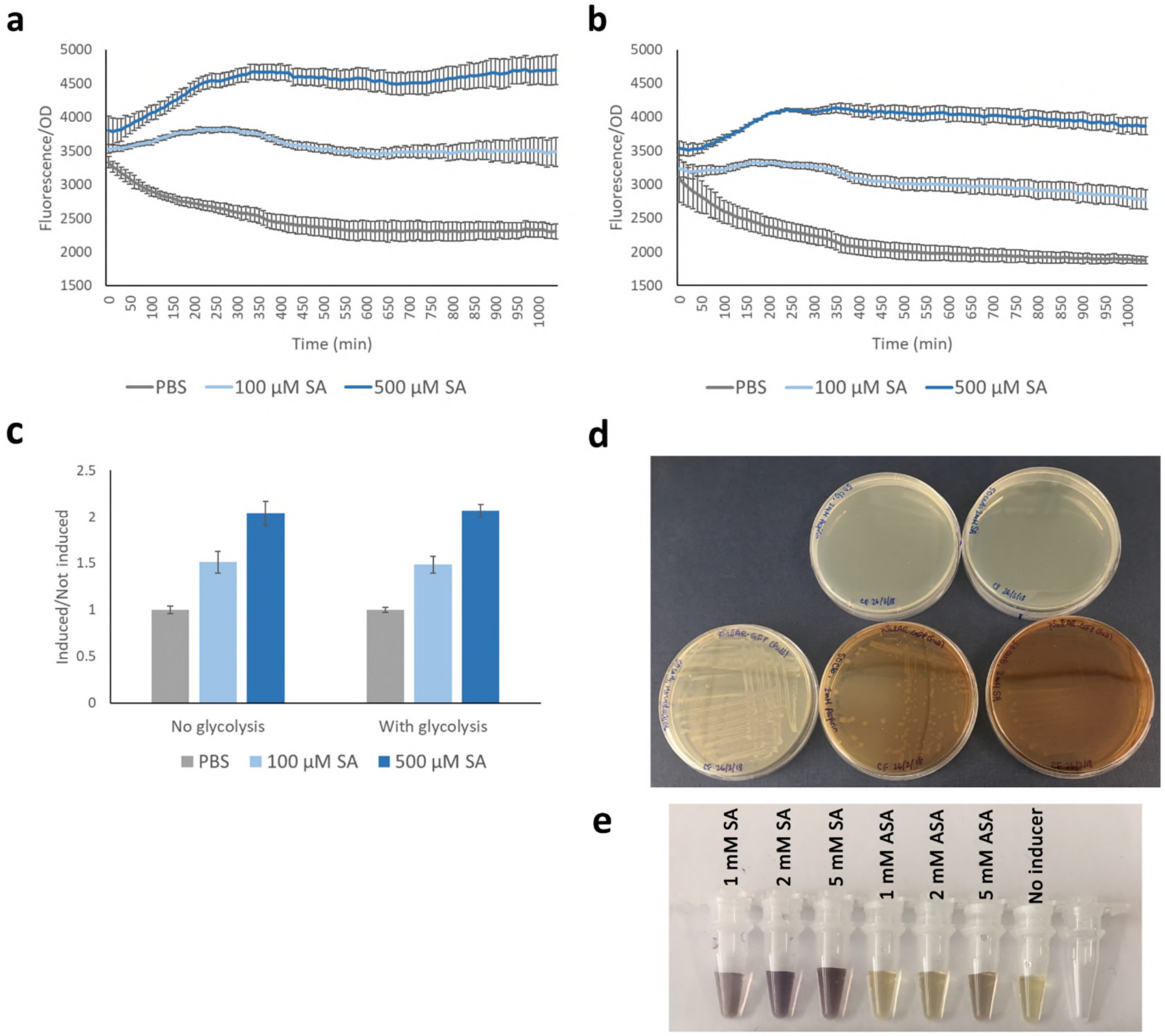

Figure S11: The levels of GFP over time after induction of the SalAR circuit can act as an indicator for the kinetics of catechol production. Production of GFP by parent cells with the pSalAR-GFP plasmid and **(a)** without an additional glycolysis pathway (pSEVA224) or **(b)** with an additional glycolysis pathway (pSEVA224-GB3). Parent cells exhibited a dosage-dependent response to SA induction. **(c)** Ratio of fluorescent output of induced cells versus cells not induced with SA. In parent cells GFP production increased by about 1.5 and 2-fold when induced with 100 µM and 500 µM SA, respectively. **(d)** Catechol production by *E. coli*. LB agar plates with aspirin and salicylic acid have no color (top), while catechol is distinctly brown (bottom). When *E. coli* with pSalAR-GFP was plated on agar with inducers 2 mM Aspirin (bottom middle) or 2 mM salicylic acid (bottom right) catechol was produced and diffused out of the cell, tinting the agar brown. The plate with no inducer did not turn brown (bottom left). **(e)** The color of the supernatant darkens as inducer concentration increases, indicating production and export of catechol. SA: salicylic acid, ASA: acetylsalicylic acid (aspirin). Data show means ± SE, n=3.

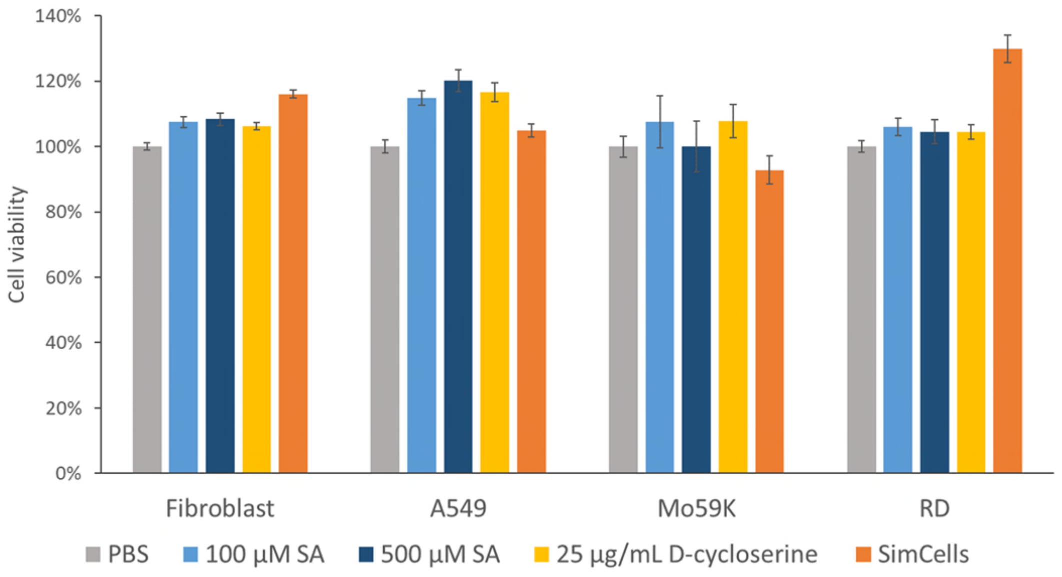

Figure S12: Cell viability of fibroblasts, A549, Mo59K, and RD cancer cells treated with supplements. These mammalian cell lines were incubated with inducer salicylic acid (SA), D-cycloserine, or SimCells. PBS was used as a control. These additions had no adverse effects on the viability of the mammalian cell lines. Data show means ± SE, n=6.

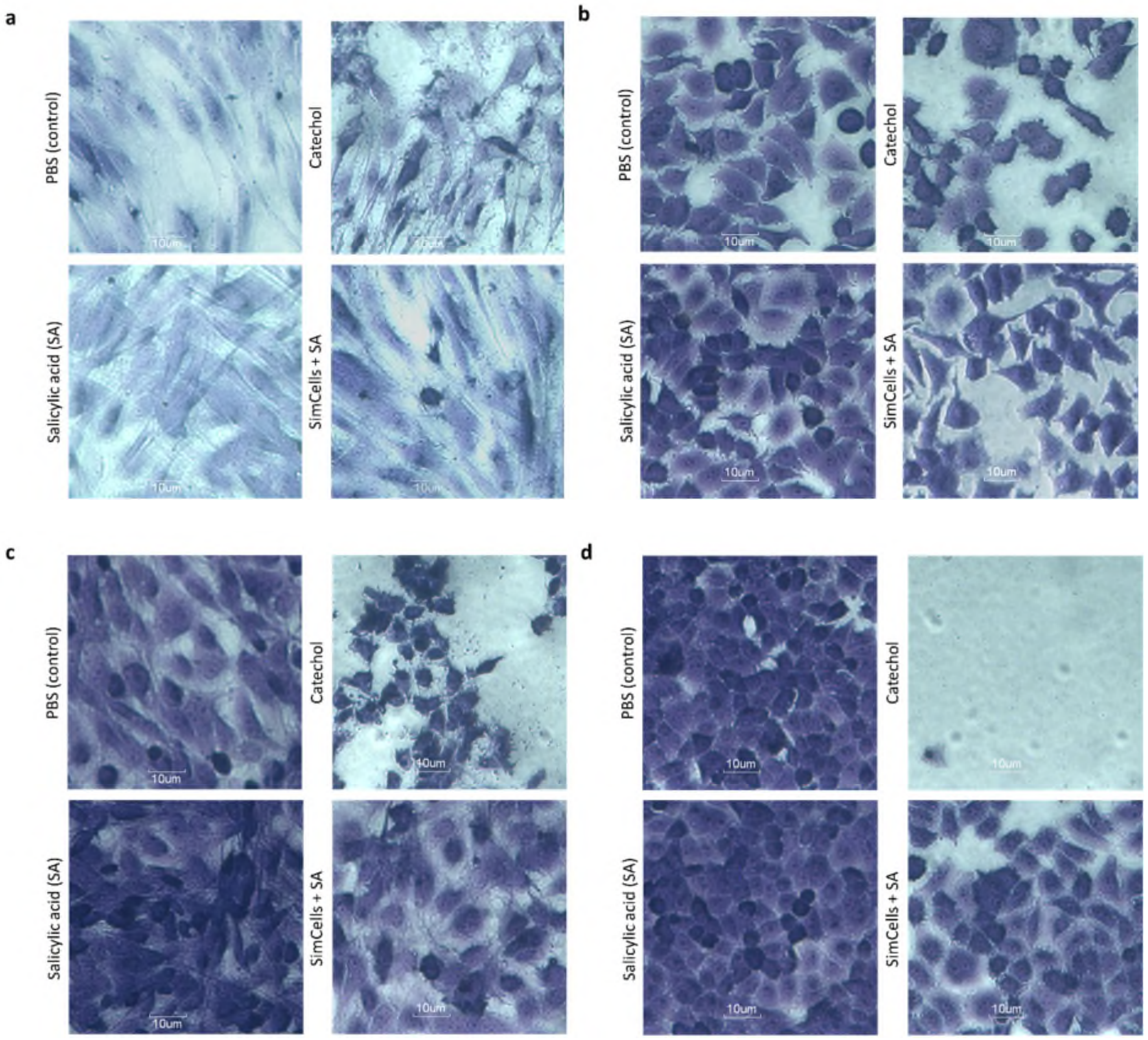

Figure S13: Brightfield images of cell lines stained with crystal violet during the cell viability assay. Cells were grown in media supplemented with PBS (control), 1 mM catechol, 500 µM salicylic acid (SA), or SimCells induced with 500 µM SA for catechol production. SA by itself had no adverse effect on cell lines. Catechol dramatically reduced cell viability of cancer cells and SimCells that synthesized catechol from SA also had some visible effect on cell viability of cancer cells. **(a)** Fibroblasts **(b)** A549 lung cancer cells **(c)** Mo59K brain cancer cells **(d)** RD soft tissue cancer cells.

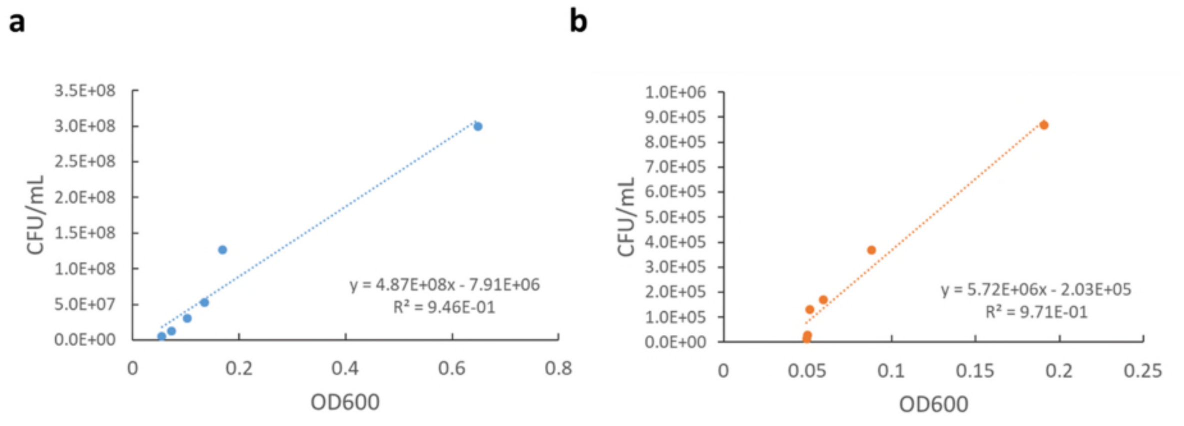

Figure S14: Relationship of OD600 and CFU/mL in (**a)** *E. coli* strain without *I-CeuI* and **(b)** with *I-CeuI*

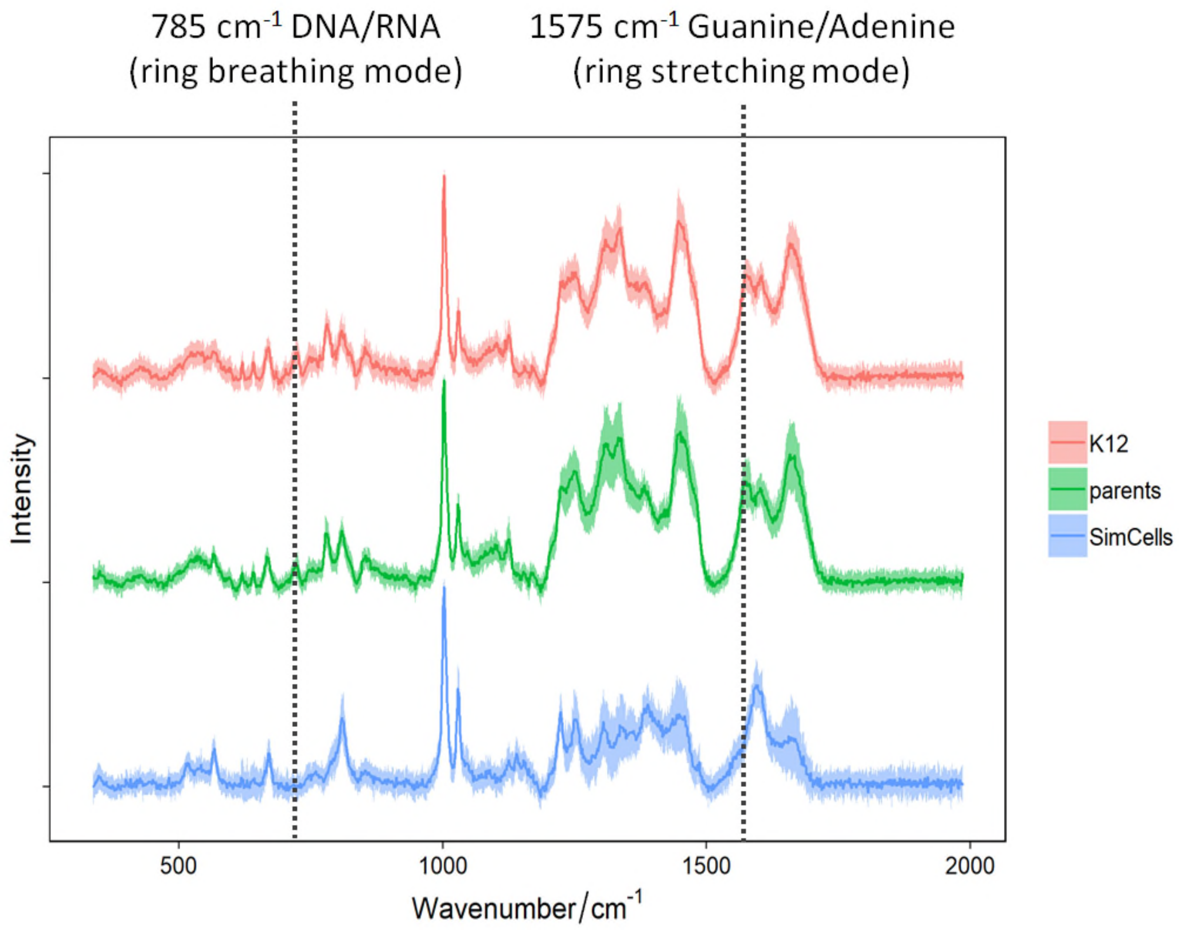

Figure S15: Raman analysis of wildtype *E. coli* K12, parent cells and SimCells. Each spectrum represents an average of single-cell Raman spectra (SCRS) from 30 single cells, and the shaded area represents the standard deviation of SCRS. SimCells displayed significantly weaker signals for characteristic Raman bands for DNA than *E. coli* K12 or parent cells.

Movie 1: Time-lapse videos shows fluorescence intensity of DAPI-stained nucleoid DNA of *E. coli* **(A)** I-CeuI+ or **(B)** I-CeuI- strains. The former took 645 min to lose fluorescence or at an estimated chromosomal degradation rate of 200 bp/sec. The latter did not decrease in fluorescence intensity.

Movie 2: Time-lapse video of *P. putida* I-CeuI+ stained with DAPI. The chromosomal DNA was lost in 450 min or at an estimated rate of 225 bp/sec.

Movie 3: Time-lapse video of *R. eutropha* I-CeuI+ stained with DAPI. The chromosomal DNA was lost in 840 min or at an estimated rate of 147 bp/sec.

Movie 4: Time-lapse video of an *E. coli* I-CeuI+ Glycolysis+ strain producing protein. Cells were stained with DAPI, which decreased in fluorescence as SimCells were generated. The chromosome-free SimCells then produced mCherry.

Movie 5: Time-lapse video of fluorescence produced by unstable mCherry with the ASV variant of the ssrA protease tag (hosted by wildtype *E. coli* I-CeuI-). After addition of this tag, mCherry-ASV has a half-life time of about 96 min.

References:

1 Zhou, L., Zhang, K. & Wanner, B. L. Chromosomal expression of foreign and native genes from regulatable promoters in Escherichia coli. *Methods Mol Biol* **267**, 123-134, doi:10.1385/1-59259-774-2:123 (2004).

2 Rogers, J. K. *et al.* Synthetic biosensors for precise gene control and real-time monitoring of metabolites. *Nucleic acids research* **43**, 7648-7660, doi:10.1093/nar/gkv616 (2015).

3 Heinemann, J. A. & Ankenbauer, R. G. Retrotransfer of IncP plasmid R751 from Escherichia coli maxicells: evidence for the genetic sufficiency of self-transferable plasmids for bacterial conjugation. *Molecular microbiology* **10**, 57-62 (1993).

4 Crameri, A., Whitehorn, E. A., Tate, E. & Stemmer, W. P. Improved green fluorescent protein by molecular evolution using DNA shuffling. *Nat Biotechnol* **14**, 315-319, doi:10.1038/nbt0396-315 (1996).

5 Sanchez-Pascuala, A., de Lorenzo, V. & Nikel, P. I. Refactoring the Embden-Meyerhof-Parnas Pathway as a Whole of Portable GlucoBricks for Implantation of Glycolytic Modules in Gram-Negative Bacteria. *ACS Synth Biol* **6**, 793-805, doi:10.1021/acssynbio.6b00230 (2017).

6 Gregor, C., Gwosch, K. C., Sahl, S. J. & Hell, S. W. Strongly enhanced bacterial bioluminescence with the ilux operon for single-cell imaging. *Proc Natl Acad Sci U S A* **115**, 962-967, doi:10.1073/pnas.1715946115 (2018).

7 Andersen, J. B. *et al.* New unstable variants of green fluorescent protein for studies of transient gene expression in bacteria. *Appl Environ Microbiol* **64**, 2240-2246 (1998).

8 Ruegg, T. L. *et al.* Jungle Express is a versatile repressor system for tight transcriptional control. *Nat Commun* **9**, doi:10.1038/s41467-018-05857-3 (2018).

9 Suomi, T., Seyednasrollah, F., Jaakkola, M. K., Faux, T. & Elo, L. L. ROTS: An R package for reproducibility-optimized statistical testing. *PLoS Comput Biol* **13**, e1005562, doi:10.1371/journal.pcbi.1005562 (2017).

10 Fan, C. *et al.* Defensive Function of Transposable Elements in Bacteria. *ACS Synth Biol* (2019).

11 Waskom, M. *et al.* mwaskom/seaborn: v0.8.1 (September 2017). doi:10.5281/ZENODO.883859 (2017).

12 Sawyer, M. & Kumar, V. A rapid high-performance liquid chromatographic method for the simultaneous quantitation of aspirin, salicylic acid, and caffeine in effervescent tablets. *Journal of chromatographic science* **41**, 393-397, doi:10.1093/chromsci/41.8.393 (2003).

13 Gregor, C., Gwosch, K. C., Sahl, S. J. & Hell, S. W. Strongly enhanced bacterial bioluminescence with the ilux operon for single-cell imaging. *Proceedings of the National Academy of Sciences of the United States of America* **115**, 962-967, doi:10.1073/pnas.1715946115 (2018).

14 Silva-Rocha, R. *et al.* The Standard European Vector Architecture (SEVA): a coherent platform for the analysis and deployment of complex prokaryotic phenotypes. *Nucleic acids research* **41**, D666-D675, doi:10.1093/nar/gks1119 (2013).

15 Ruegg, T. L. *et al.* Jungle Express is a versatile repressor system for tight transcriptional control. *Nature Communications* **9**, doi:10.1038/s41467-018-05857-3 (2018).

16 Huang, W. E. *et al.* Chromosomally located gene fusions constructed in Acinetobacter sp. ADP1 for the detection of salicylate. *Environmental microbiology* **7**, 1339-1348, doi:10.1111/j.1462-5822.2005.00821.x (2005).

17 Huang, W. E., Singer, A. C., Spiers, A. J., Preston, G. M. & Whiteley, A. S. Characterizing the regulation of the Pu promoter in Acinetobacter baylyi ADP1. *Environmental microbiology* **10**, 1668-1680, doi:10.1111/j.1462-2920.2008.01583.x (2008).
